# Age-related physiological dysregulation progresses slowly in semi-free-ranging chimpanzees

**DOI:** 10.1093/emph/eoae010

**Published:** 2024-06-19

**Authors:** Megan F Cole, Paige Barnes, Isabelle G Monroe, Joshua Rukundo, Melissa Emery Thompson, Alexandra G Rosati

**Affiliations:** Department of Anthropology, University of New Mexico, Albuquerque, NM, USA; Department of Psychology, University of Michigan, Ann Arbor, MI, USA; Department of Psychology, University of Michigan, Ann Arbor, MI, USA; Chimpanzee Sanctuary and Wildlife Conservation Trust, Entebbe, Uganda; Department of Anthropology, University of New Mexico, Albuquerque, NM, USA; Department of Psychology, University of Michigan, Ann Arbor, MI, USA; Department of Anthropology, University of Michigan, Ann Arbor, MI, USA

**Keywords:** primates, health, aging, lifestyle, human evolution

## Abstract

**Background and objectives:**

Lifestyle has widespread effects on human health and aging. Prior results from chimpanzees (*Pan troglodytes*), one of humans’ closest evolutionary relatives, indicate that these lifestyle effects may also be shared with other species, as semi-free-ranging chimpanzees fed a naturalistic diet show healthier values in several specific health biomarkers, compared with their sedentary, captive counterparts. Here, we examined how lifestyle factors associated with different environments affect rates of physiological aging in closely related chimpanzees.

**Methodology:**

We compared physiological dysregulation, an index of biological aging, in semi-free-ranging chimpanzees in an African sanctuary versus captive chimpanzees in US laboratories. If the rate of aging is accelerated by high-calorie diet and sedentism, we predicted greater age-related dysregulation in the laboratory populations. Conversely, if costs of a wild lifestyle accelerate aging, then semi-free-ranging chimpanzees at the sanctuary, whose environment better approximates the wild, should show greater age-related dysregulation. We further tested whether dysregulation differed based on sex or body system, as in humans.

**Results:**

We found that semi-free-ranging chimpanzees showed lower overall dysregulation, as well as lower age-related change in dysregulation, than laboratory chimpanzees. Males experienced lower dysregulation than females in both contexts, and the two populations exhibited distinct aging patterns based on body system.

**Conclusions and implications:**

Our results support the conclusion that naturalistic living conditions result in healthier aging in chimpanzees. These data provide support for the proposal that lifestyle effects on human health and aging are conserved from deeper into our evolutionary history.

## INTRODUCTION

Lifestyle factors have widespread effects on human health and longevity. For example, sedentism, high-calorie diets and smoking predict incidence of chronic disease (e.g. cardiovascular disease, diabetes and cancer) [1, 2] as well as acceleration of some biological measures of aging, such as telomere length and oxidative stress [3, 4]. These lifestyle factors may explain some population differences. For example, small-scale subsistence populations, which have high levels of physical activity and diets low in processed foods, display lower rates of cardiovascular disease compared to industrialized human populations [5, 6]. This is in contrast to the lower adult life expectancies in these populations, which may be influenced by comparatively high levels of infectious disease [7, 8]. It is less clear how these factors may influence rates of biological aging across populations. Tsimane forager-horticulturalists of the Bolivian Amazon, who experience a high infectious disease burden but also physically active lifestyles, exhibit a slightly higher rate of physiological aging compared with a combined industrialized sample [9] but appear to age more slowly than White Americans by some epigenetic measures [10]. While comparative demography supports the conclusion that aging patterns may be somewhat constrained at the species level [11], environmental factors may play a strong role in determining ‘healthy aging’, the resistance to disease and disability with age. To better understand how lifestyle differences affect aging in long-lived species, we examined how analogous lifestyle factors associated with different living environments affect age-related physiological dysregulation in chimpanzees (*Pan troglodytes*), one of humans’ two closest living relatives. Captive chimpanzees live significantly longer than most chimpanzees in the wild but are more likely to die of chronic (e.g. heart) disease, while wild mortality is more often linked to infectious (e.g. respiratory) disease or injury [7]. Notably, prior work suggests that chimpanzees in naturalistic living conditions that more closely approximate the wild also show improvements in some biomarkers of health, such as weight and cholesterol [12, 13]. As these effects are analogous to those observed between industrialized and subsistence human populations, comparisons across chimpanzee populations living in different environments provide an informative model for understanding the evolutionary history of lifestyle effects on human health.

Aging is a complex process involving changes across multiple interacting systems, posing challenges for comparative research. Complex systems approaches, such as quantifying multisystem physiological dysregulation, provide a holistic basis for comparing aging across individuals, populations or even species [14]. Physiological dysregulation is an emergent phenomenon that reflects the breakdown of physiological regulatory networks’ ability to maintain homeostasis and is implicated as both a consequence and driver of the aging process [14–16]. Dysregulation can be quantified using the statistical index *Mahalanobis distance* (*D*_*M*_), which calculates the distance of a set of biomarkers from a healthy baseline, typically a young adult population average [17, 18]. In particular, higher *D*_*M*_ scores reflect biomarker profiles that are more divergent from baseline or involve more unusual combinations of biomarker concentrations, indicating greater disruption of homeostasis. In humans, *D*_*M*_ reliably increases with age—and often exponentially during old age—and is predictive of mortality and health outcomes such as frailty, cardiovascular disease and diabetes [17, 19–21]. Importantly, this index is broadly comparable across study systems even when different biomarkers are included or when differing laboratory methods were used to assess them [17, 22]. Although this approach focuses on including biomarkers that should be independent, the *D*_*M*_ metric automatically adjusts for the correlation structure among biomarkers, and analyses can further account for the total number of biomarkers used. Importantly, this multivariate measure is by design not subject to substantial influence by any one single variable, allowing it to better capture high-order regulatory processes compared to any individual biomarker [23]. As such, physiological dysregulation has emerged as a useful and holistic metric of aging and associated health decline in humans.

The *D*_*M*_ method is also a valuable tool for comparative studies of aging across populations and even across species. Comparative data demonstrate that the age-associated increase in *D*_*M*_ is a conserved signal of aging that is detectable across different human populations [19, 21], as well as across primate taxa [24]. However, in a cross-species analysis of 11 primate species, Dansereau *et al.* [24] found significant species variation associated with lifespan and phylogenetic distance. Humans, the longest-lived species, exhibited the least amount of age-related change in *D*_*M*_ (i.e. the lowest age slope), consistent with our exceptionally slow pace of aging [25, 26]. Physiological dysregulation in chimpanzees, who are also long-lived, increased markedly less steeply across the lifespan in comparison to other primate taxa like monkeys and lemurs with faster life histories. Indeed, chimpanzees and humans showed the greatest comparability in patterns of dysregulation across this sample.

Prior work has also highlighted that physiological dysregulation may differ between sexes within the same species. Sex differences in aging patterns in humans are generally consistent with the male–female health-survival paradox, where females live longer than males despite experiencing poorer health across the lifespan [27]. Accordingly, Arbeev *et al.* [28] found faster dysregulation in human females, but higher mortality risk at a given *D*_*M*_ in males. In contrast, other work [29] has found no sex differences in dysregulation during aging in humans. Available data on sex differences in physiological dysregulation in captive primates is mixed [24], so it is not currently clear if these sex differences in dysregulation patterns are more widely shared.

Finally, recent research shows that physiological dysregulation can proceed at different rates or have differential health impacts across different body systems. For instance, Li *et al.* [23] found that while *D*_*M*_ was correlated across six body systems and generally increased with age (except in lipids), these systems differed in their ability to predict health outcomes. For example, whereas all systems reliably predicted mortality, cardiovascular disease incidence was associated only with liver and vitamin dysregulation, and diabetes incidence was associated only with electrolyte, liver and lipid dysregulation. This study provides a strong rationale for measuring integrated, multi-system physiological dysregulation and supports the hypothesis that aging is a suite of emergent, interdependent processes, rather than either a single global process or entirely independent processes in different systems [14, 23].

One limitation of existing studies of physiological dysregulation is that they have been mostly restricted to humans living in industrialized societies and captive primates (but see Refs. [30, 31]). As a result, these data may not be broadly representative of physiological aging in these species. For example, many of the captive primates studied in prior work live in laboratories or other species-atypical environments, while the industrialized environments many human populations live in today depart significantly from the contexts that shaped the evolution of our species [32]. Some work has addressed this by extending studies of physiological dysregulation cross-culturally to the Tsimane, a population of Bolivian forager-horticulturalists [9]. This population is notable for their healthy lifestyle and low risk of cardiovascular disease [5], but also for living in harsh environmental conditions, characterized by high pathogen load and high energy expenditure [8, 33]. The Tsimane exhibited dysregulation scores 2–12 times lower than most primates (with one exception being male chimpanzees), but only about 15% higher than industrial humans. While the different methods preclude direct comparison across these studies, these data suggest that human dysregulation is fairly conserved at the species level but is also still sensitive to environmental context. Furthermore, healthy aging (i.e. low incidence of chronic disease during old age) may not necessarily be associated with slower physiological aging. This highlights the importance of understanding how lifestyle factors affect different components of the aging phenotype in humans and other long-lived primates.

Comparative data from chimpanzees—who share much of our evolutionary history, a long lifespan and genetic and physiological similarities—can inform biological perspectives on human health and aging by helping to identify processes that may have shaped our species’ health in our evolutionary past, as well as to understand more broadly how health and aging characteristics differ across species [34, 35]. In the current study, we examined patterns of physiological dysregulation in primarily wild-born, semi-free-ranging chimpanzees living in an African sanctuary, comparing them to available data on captive chimpanzees living in US laboratories. African sanctuary chimpanzees have access to species-appropriate forest habitats and eat a naturalistic diet dominated by fruits and vegetables [36]. This contrasts with the relatively sedentary lifestyle of laboratory chimpanzees, who consume mainly processed foods. African sanctuary chimpanzees notably show species-typical patterns of behavior and physiology [37, 38]. They exhibit cardiovascular profiles characterized by lower body weight, lower blood pressure and healthier levels of blood lipids than chimpanzees living in US laboratories [12, 39, 40], as well as hormonal and viral infection patterns similar to their wild counterparts [41–43].

In comparing age-related dysregulation in chimpanzees across contexts, we aimed to test between several alternative hypotheses about how lifestyle shapes the rate of aging. First, if the rate of aging is accelerated by high-calorie diet and sedentary lifestyle, we predicted greater age-related dysregulation in chimpanzees living in laboratories. In contrast, if the demands associated with living in the wild—which might include exposure to species-typical pathogens or the costs associated with more intense physical activity in such environments [44, 45]—increases the rate of aging, then we would expect greater age-related dysregulation in sanctuary chimpanzees, whose environments better approximate the wild. Finally, it is also possible that there is no major difference in age-related dysregulation at the sanctuary versus laboratories, for example, because the rate of aging in chimpanzees may not be particularly sensitive to lifestyle, or if the shared features of both environments (such as routine veterinary care) result in comparable rates of aging in both populations. We further tested whether dysregulation differed based on sex or body system, as observed in humans, across these two populations, with the goal of examining if chimpanzees share some of these more nuanced features of human aging.

## METHODS

### Sanctuary chimpanzee data and ethics statement

Research at Ngamba Island Chimpanzee Sanctuary was approved by the Uganda Wildlife Authority, the Uganda National Council for Science and Technology, and the Institutional Animal Care and Use Committee at the University of Michigan, the University of New Mexico and Harvard University. Research practices and animal care procedures complied with the Pan-African Sanctuary Alliance standards, and shipment of chimpanzee blood complied with international CITES regulations. Data collected for this project are available from the Dryad Digital Repository: https://doi.org/10.5061/dryad.37pvmcvt7. The comparison dataset is available from the Primate Aging Database at https://primatedatabase.org/.

For this project, we curated health data from 46 healthy adult chimpanzees living at the sanctuary (see Table 1 for description of sanctuary versus laboratory chimpanzees). This included 27 females and 19 males ranging in age from 15 to 38 years (mean = 24.1). All of the sanctuary chimpanzees were socially housed, with semi-free-ranging access to ~40 hectares of tropical forest (some individuals may be retained separately from the group due to health or husbandry issues, during which time they may have access to smaller enclosures). These chimpanzees can forage on wild foods in their forest enclosures, and their diet was supplemented with species-appropriate fruits and vegetables. Importantly, this provisioning does not include any high-calorie primate chow typical of captive chimpanzee diets. Most sanctuary individuals are wild-born orphans who were mother-reared in the wild for 1–3 years and integrated into species-typical social groups upon arrival at the sanctuary (one subject in this study was born at the sanctuary due to contraception failure). Age was estimated by sanctuary veterinarians on arrival and validated by patterns of dental emergence and body weight during subsequent health checks [12, 46].

**Table 1.** Lifestyle factors of sanctuary versus laboratory chimpanzees

	Sanctuary	Laboratories
Location	Ngamba Island Chimpanzee Sanctuary, Uganda	3 US laboratories from NIH Primate Aging Database
Housing	Mixed-age and -sex grouping(s)	‘Social’
Diet	Wild foods supplemented with domesticated fruits and vegetables; no primate chow	‘Only chow’ or ‘Chow supplemented with fruits and vegetables’
Ranging	Semi-free ranging in large primary rainforest enclosures	‘Indoors’, ‘Outdoors’ or ‘Mixed’
Age	15–38 years (mean = 24.1)	15–38 years (mean = 23.5)
*N*	46 adults (27 females) 392 health checks	325 adults (188 females) 3351 health checks

Diet and physical activity patterns at African sanctuaries more closely approximate wild contexts. Laboratory lifestyle categories were extracted from the Primate Aging Database at the site level.

All data for sanctuary chimpanzees were collected during routine annual health examinations conducted by sanctuary veterinarians between 2012 and 2022. These biomarkers were compiled from several sources. First, every year, body weight was measured using a scale and blood samples were taken for standard testing at local medical clinics. This typically included complete blood count tests that assess the health and function of the circulatory system (e.g. platelets, hemoglobin), the liver and kidney system (e.g. bilirubin, albumin) and the immune system (e.g. white blood cell counts). Because samples were assayed at several different local clinics over the course of the data collection, we ensured that data were comparable within and between years by converting individual biomarker units as necessary and visually checking that reference ranges were consistent across time (see supplemental information for details). Second, for two of the sampled years, we included several additional biomarkers of the cardiometabolic system obtained from samples collected for specific research projects. Specifically, we measured skinfold thickness (as a metric of adiposity) and several cardiovascular biomarkers (e.g. cholesterol, triglycerides) on-site using a portable Alere Cholestech LDX System (see Cole *et al.* [12], for details of the lipid sampling). We further assayed samples collected in those years for biomarkers of inflammation and oxidative stress at the University of New Mexico Comparative Human and Primate Physiology Center. For inflammation, we measured C-reactive protein (CRP, Catalog No. KHA0031, Thermo Fisher Scientific), Interleukin 6 (IL-6, Catalog No. BMS213HS, Thermo Fisher Scientific), and urinary neopterin (Ref. RE59321, IBL International). For oxidative stress, we measured urinary 8-hydroxy-2'-deoxyguanosine (OHdG, Catalog No. KOG-200S/E, Genox JaICA), 15-Isoprostane F_2t_ (isoprostanes, Prod. No. E85, Oxford Biomedical Research), and total antioxidant capacity (TAC, Item No. 709001, Cayman Chemical) (see Thompson González *et al.* [47] for details of assay methods). We excluded data from chimpanzees who were known to be ill at the time of sampling (i.e. where veterinary notes or clinical results indicated clear illness at the time of sample collection; *N* = 4 datapoints from four chimpanzees).

The sanctuary dataset spanned 11 years of longitudinal data for a total of *N* = 392 chimpanzee datapoints. This included up to 50 biomarkers per health check comprising four body systems: circulatory (12 biomarkers); liver and renal (16 biomarkers); immune (12 biomarkers) and cardiometabolic (10 biomarkers) (see Table 2 for all biomarkers included in the dataset sorted by body system and chimpanzee population; Supplementary Table S1 for details about how these biomarkers were statistically transformed by site, as described in more detail below; and supplemental excel files for correlation matrices for biomarkers at each site).

**Table 2.** Biomarkers included in *D*_*M*_ calculation, by body system

Circulatory	Liver and renal		Immune	Cardiometabolic
Red blood cell count (RBC)^C^	Creatinine (CR)^C^	Alanine transaminase (ALT)^S^	White blood cell count (WBC)^C^	Body weight^C^
Hemoglobin (HGB)^C^	Globulin (GLOB)^C^	Direct bilirubin (DBIL)^S^	Lymphocytes (LYM)^C^	Glucose (GLU)^C^
Hematocrit (%HCT)^C^	Alkaline phosphate level (ALP)^C^	Indirect bilirubin (IBIL)^S^	Monocytes (MON)^C^	Total cholesterol (TC)^C^
Mean corpuscular volume (MCV)^C^	Sodium (NA)^C^	Calcium (CA)^L^	Eosinophils (EOS)^C^	Triglycerides (TRG)^C^
Mean corpuscular hemoglobin (MCH)^C^	Potassium (K)^C^	Creatinine phosphokinase (CPK)^L^	Basophils (BAS)^C^	TC/HDL ratio
Mean corpuscular hemoglobin concentration (MCHC)^C^	BUN/creatinine ratio (BUN/CR)^C^	Lactic dehydrogenase (LDH)^L^	C-reactive protein (CRP)^S^	Inguinal skinfold thickness^S^
Platelet count (PLT)^C^	Albumin/globulin ratio (ALB/GLOB)^C^	Glutamic pyruvic transaminase (SGPT)^L^	Interleukin 6 (IL6)^S^	Biceps skinfold thickness^S^
Red blood cell distribution width (%RDW)^S^	Albumin/protein ratio (ALB/TP)^C^	Glutamic oxalocetic transaminase (SGOT)^L^	Neopterin^S^	Triceps skinfold thickness^S^
Men platelet volume (MPV)^S^	Total bilirubin (TBIL)^C^	Phosphorous (PO4)^L^	8 Hydroxy deoxyguanosine (OHdG)^S^	Subscapular skinfold thickness^S^
Platelecrit (%PCT)^S^	Gamma-glutamyl (GGT)	Uric acid (UA)^L^	Isoprostanes^S^	Upper arm circumference^S^
Platelet distribution width (%PDW)^S^	Chloride (CL)	Magnesium (MG)^L^	Total antioxidant capacity (TAC)^S^	
Platelet larger cell ratio (%P-LCR)^S^	Bicarbonate (HCO3)	Lipase (LIPA)^L^	Neutrophils (NEU)^S^	
Iron (FE)^L^	Asparate aminotransferase (AST)^S^	Amylase (AMYL) ^L^	Neutrophils banded (NEU-B)^L^	
			Neutrophils segmented (NEU-S)^L^	

Biomarkers were in both the sanctuary dataset and the laboratory datasets (i.e. available for one or more laboratory sites), unless denoted S (only at sanctuary; *N* = 21) or L (only at laboratories; *N* = 13). C indicates biomarkers available at all four sites (*N* = 25). Main analyses used all available biomarkers at each site.

### Laboratory chimpanzee data

We extracted a comparative dataset for laboratory-living chimpanzees from the Primate Aging Database (accessed February 2021). This publicly available database comprises biomarker data, collected quarterly, from healthy, non-experimental captive chimpanzees living in three US laboratory sites: Alamogordo Primate Facility, Yerkes National Primate Research Center, and MD Anderson Cancer Center. Based on information provided in the Primate Aging Database, these chimpanzees were socially housed (categorized broadly at the site level as indoors; outdoors; or some outdoor access) and fed a diet of primarily primate chow (categorized as only chow; or chow supplemented with fruits and vegetables) [48]. We converted each biomarker as necessary such that units matched the sanctuary data, and further ensured that data were comparable across sites by visually checking that distributions overlapped with one another and human reference ranges.

Because the full laboratory dataset encompassed a larger age range compared to Ngamba (up to 58.25 years), our main analyses used a truncated, age-matched dataset of up to 38 years across groups. The main laboratory dataset comprised *N* = 3351 datapoints from 325 chimpanzees (188 females and 137 males; mean age = 23.5). This included up to 42 biomarkers per health check, representing the same four body systems as at Ngamba: circulatory (8 biomarkers); liver and renal (22 biomarkers); immune (7 biomarkers) and cardiometabolic (5 biomarkers) (see Table 2 and Supplementary Table S1 for details). Twenty-nine of the biomarkers available at the laboratories were direct matches to those at the sanctuary—specifically from complete blood count tests. Additional variables unique to the laboratories included other common indices of liver and renal function (minerals and enzymes such as iron and phosphorous).

### *Biomarker selection and* Mahalanobis distance (D_M_) *calculation*

Because the sanctuary dataset sometimes included multiple measurements from the same chimpanzee in close succession (e.g. if an individual was being monitored for active illness or injury), we restricted data to measurements at least 0.25 years apart per individual to match the rate of sampling in the Primate Aging Database. In order to ensure we had sufficient longitudinal data from a given individual, we further required that each chimpanzee in each dataset have measurements from at least two time points (i.e. two different health checks) per biomarker, and at least five biomarkers per time point. The sample sizes listed above were calculated after these exclusions.

Before calculating *D*_*M*_, we removed outliers, such as biologically implausible values or extreme statistical outliers (see supplemental information for details). We then standardized each biomarker separately for each site. Specifically, all individual biomarkers were log- or square-root-transformed as relevant for the reference sample to approach normality and were then *z*-scored to the reference sample [17, 18] (see Supplementary Table S1 for a detailed list of data transformations for each biomarker by site). We used all available biomarkers at each site in the main analyses.

*D*_*M*_ is calculated as:


$$\begin{array}{l} D_{m}=\sqrt{{\left(x-\mu \right)}^{T}S^{-1}(x-\mu ),} \end{array}$$

where *x* is a multivariate observation, µ is the mean of the reference sample, and *S* is the variance–covariance matrix of the reference sample; *T* indicates the transpose of the matrix [18]. Given that this method accounts for correlation structure, it not only allows variables to be highly correlated, but it also corrects for any potential redundancy among variables. This is important when modeling emergent processes, where multiple biomarkers may feed back onto one another [23]. Due to uneven sampling across time, we calculated *D*_*M*_ using the *MDmiss* package in R, which accounts for missing observations [9]. Because *MDmiss* requires no missing values in correlation matrices, and removing all observations with missing values from the references would have greatly reduced the sample sizes, we followed the approach described by Kraft *et al.* [9] of populating missing values in reference correlation matrices as the medians of each biomarker from each reference sample. *MDmiss* produces a square of *Mahalanobis distance*, so we took the square root to obtain raw *D*_*M*_ scores. We used the logarithm of *D*_*M*_ for analyses, *z*-scored (centered to the mean and divided by the standard deviation) within each site.

To accurately measure changes in dysregulation in each population of chimpanzees, our primary analyses used a site-specific reference sample to calculate *D*_*M*_ separately for each of the four sites (one sanctuary and three laboratories). This approach allowed us to examine age-related changes at each site while also accounting for any baseline physiological differences across groups. Following best practices, reference samples comprised data from healthy young adults. This included non-overweight individuals aged 15–20 years old [49]. While some approaches for calculating this reference sample focus on using only the first measurement (e.g. the youngest visit) of a given individual [17, 24], this would have provided a reference sample size that was insufficient to analyze the current data. Following the recommendation of Cohen *et al.* [22], we, therefore, used all data (e.g. including repeated measurements) of individuals who fell in this young, non-overweight range.

We used a weight cutoff to calculate the reference samples because overweight and obesity are associated with negative health outcomes such as elevated blood pressure and proatherogenic blood lipids [12, 50, 51], and as such is an informative proxy for health status. We categorized non-overweight females as ≤54.4 kg following Videan *et al.* [51], where 54.4 kg corresponded to BMI 20% above the group mean in a population of laboratory-housed chimpanzees (and we could not calculate BMI in the current study). This value has subsequently been used as an overweight threshold in other captive chimpanzee studies [50]. We confirmed this was appropriate in the current study by visually assessing this cutoff in our pooled data (combining all three laboratories as well as Ngamba) and determined that including weights above 54.4 kg shifted a relatively normal distribution to a right skewed, bimodal distribution (Supplementary Fig. S1). We applied the same methodology to the male data, where published standards were unavailable, to categorize non-overweight as ≤67.4 kg. We confirmed this approach was not too conservative by additionally using the upper quintile (top 20%) of sex-specific weight measurements as an overweight cutoff (68 kg for females and 70 kg for males) (see supplemental information for details).

### Statistical analysis

We asked whether age-related change in *D*_*M*_ varied by (1) *facility* (sanctuary or laboratory), (2) *sex* (female or male), and (3) *body system* (circulatory; liver and renal; or immune), using linear mixed effects models implemented with the *lmer* function in R. Our general approach was to first construct a base model with a random effect of *subject identity* to account for unbalanced repeated measurements across multiple timepoints, and fixed effects of *age* (in years) and *number of biomarkers* (at that measurement timepoint) [9, 17]. Because number of biomarkers is generally positively correlated with overall dysregulation scores, it is standard practice to account for this as a control variable during statistical modeling. In each set of models, we then tested whether the independent variable of interest significantly affected *D*_*M*_, alone or in interaction with age, using likelihood ratio tests. We finally conducted post hoc analyses using the *emtrends* function to examine pairwise comparisons of age-related trends.

In our first analysis, which tested the effect of *facility* and *age* on the rate of dysregulation, we directly compared age-related change in *D*_*M*_ in the sanctuary versus laboratory datasets. In contrast, we examined the impacts of *sex* and *body* system separately in the sanctuary and laboratory populations. While *D*_*M*_ values were calculated separately for each population to account for any differences in baseline health profiles, we pooled these standardized values in the analyses to focus on the broad comparison between laboratory and sanctuary. In laboratory-only models, we controlled for *site* location.

The rate of age-related dysregulation can be quantified via the age slope of *D*_*M*_, or its standard effect coefficient for *age* [23]. We, therefore, report age slopes from mixed models for each *facility*, *sex* and *body system* (with the latter two calculated separately at each facility). This value can be used to compare rates of aging across populations or predictors *within* a study (e.g. whether sanctuary versus laboratory chimpanzees exhibit different rates of aging across body systems) but also *between* studies (e.g. whether global dysregulation in sanctuary chimpanzees progresses more rapidly than in human samples). To this end, we used a standardized (*z*-scored) metric of age throughout to facilitate direct comparison across studies, regardless of the age range sampled [9, 24]. However, figures use unstandardized ages for ease of interpretation.

We further conducted several data checks to ensure our results were methodologically robust since dysregulation scores can be sensitive to selection of reference samples and biomarker composition [22, 24]. First, we ran the primary analysis (i.e. comparing dysregulation in sanctuary and laboratory chimpanzees) using the full age range at the laboratories (up to 58.25 years), to assess potential increases in the rate of aging during senesence. This also was important since slopes reported from prior studies using Primate Aging Database data did not use an age cutoff. Second, we ran these primary analyses using a pooled reference sample (which included all reference observations across all four sites), to examine if the choice of the reference sample drove our primary results. Third, we ran these analyses using a set of 25 common biomarkers (which were present at all four sites), rather than the full set of available biomarkers, to examine how biomarker selection may have impacted results. Finally, as noted above we checked if a different overweight cutoff for the healthy reference sample impacted these patterns.

## RESULTS

We first examined the data to ensure it met assumptions of the approach. Initial visualization indicated that raw *D*_*M*_ scores increased with age in laboratory and sanctuary chimpanzees (Supplementary Fig. S3). This validates that the *D*_*M*_ method captures age-related physiological dysregulation in both populations in the current study. Many individual biomarkers were weakly but significantly associated with age (See Supplementary Table S2 for biomarker correlations to age and *D*_*M*_ as well as supplemental excel files for pairwise correlation matrices of individual biomarkers at each site). Only one was highly correlated with *D*_*M*_ (|*r*|> = 0.70): banded neutrophil count (NEU-B) at Yerkes, which we removed in the primary analyses [17]. However, this removal did not appreciably alter the pattern of results. Overall, this suggests that our calculations of *D*_*M*_ reflect a holistic measure of health that are not dependent on any one biomarker.

**Figure 1.**
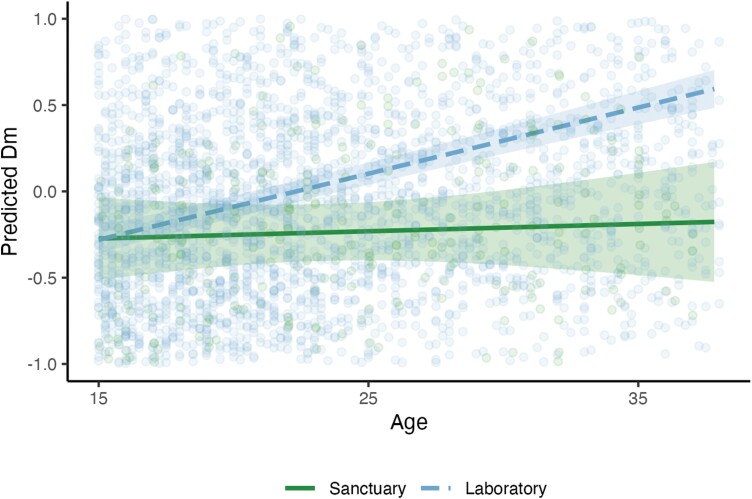
Age-related change in *D*_*M*_, split by *facility*. Predicted values are estimates of *D*_*M*_ (log-transformed and *z*-scored within each site) from model accounting for *subject identity, age*, *facility*, *age* × *facility* and *number of biomarkers*. Lines indicate linear fit; ribbons indicate 95% confidence intervals. See Supplementary Fig. S3 for raw dysregulation scores, which increase in both groups

We first modeled whether *D*_*M*_ differed across the sanctuary versus laboratory populations (*N* = 392 and 3351 datapoints, respectively; Fig. 1; Supplementary Table S3). *D*_*M*_ increased with *age* in the base model [*ß* = 0.22; *P* < 0.001], Adding *facility* as a predictor improved model fit [*χ*^2^ = 8.49, df = 1, *P* < 0.01]: *D*_*M*_ was higher overall at the laboratories, indicating that these populations of chimpanzees exhibited greater levels of dysregulation from homeostasis. Subsequently adding *age* × *facility* also improved model fit [*χ*^2^ = 9.51, df = 1, *P* < 0.01]. Post hoc analysis revealed that *D*_*M*_ increased with age faster in the laboratory populations (*P* < 0.01). Notably, model-predicted values of *D*_*M*_ in the *facility* analysis comparing both sanctuary and laboratory populations were generally negative at Ngamba, despite raw scores being *z*-scored within each site. This is likely because the predicted values account for the effect of *facility* on these scores, as well as group differences in *number of biomarkers* (which was typically much higher at Ngamba). We compared the effect of age on *D*_*M*_ in sanctuary versus laboratory chimpanzees by extracting the standard effect coefficient for age in separate linear mixed effects models for each facility controlling only for *subject identity* and *number of biomarkers* (and *site* for the laboratory model), that is, without accounting for *age* × *facility* interaction. Age slopes were much steeper at the laboratories than at Ngamba (0.24 compared to 0.18; see Table 3 for age slopes from each model).

**Table 3.** Standardized age slopes (95% confidence interval) calculated separately by *facility*, *sex* and *body system*

	Sanctuary	Laboratories
*Facility* models	**0.18 (−0.01, 0.30)**	**0.24 (0.21, 0.28)**
*Sex* models		
Females	0.13 (−0.12, 0.26)	**0.21 (0.17, 0.26)**
Males	**0.28 (0.11, 0.41)**	**0.27 (0.20, 0.34)**
*Body system* models		
Circulatory system	−0.02 (−0.16, 0.10)	**0.06 (0.02, 0.11)**
Liver and renal system	**0.18 (0.05, 0.30)**	**0.18 (0.14, 0.22)**
Immune system	**0.13 (0.02, 0.23)**	**−0.05 (−0.09, −0.01)**
Cardiometabolic system	**0.33 (0.06, 0.61)**	−0.04 (−0.30, 0.23)
Supplemental *facility* models		
Full laboratory age range	*NA*	**0.24 (0.21, 0.28)**
Pooled reference	0.03 (−0.12, 0.12)	**0.19 (0.15, 0.23)**
Common biomarkers	**0.16 (0.02, 0.28)**	**0.30 (0.26, 0.34)**
Alternative overweight cutoff	**0.17 (−0.01, 0.28)**	**0.23 (0.20, 0.27)**

Age slopes are standard effect coefficients for *age* (*z*-scored within each site) from models controlling for *subject identity* and *number of biomarkers* (and *site* for laboratory models). Unlike in the main analysis, models were run separately for each *facility*, *sex* (for each facility) and *body system* (for each facility) to allow for comparison of age effects on dysregulation between and within datasets. Facility, sex and body system models used the main dataset. Supplemental models used different datasets for each analysis, which are described in more detail in the supplemental information. Bolded slopes were significantly different from zero.

**Figure 2.**
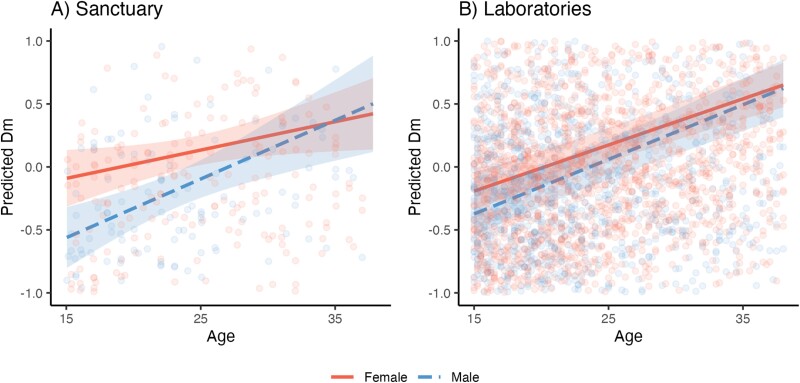
Age-related change in *D*_*M*_, split by *sex* at the (A) sanctuary and (B) laboratories. Predicted values are estimates of *D*_*M*_ (log-transformed and *z*-scored within each site) from models accounting for *subject identity, age*, *sex*, *age* × *sex* and *number of biomarkers* (and *site* for the laboratory model). Lines indicate linear fit; ribbons indicate 95% confidence intervals. See Supplementary Fig. S4 for raw dysregulation scores

We next examined sex differences in dysregulation in sanctuary-living and laboratory-living chimpanzees, conducting these analyses separately for each population (Supplementary Fig. S4; Fig. 2; Supplementary Table S4). Consistent with the above results, the age effect was positive in the sanctuary base model [*ß* = 0.18; *P* < 0.01], indicating that *D*_*M*_ increased with age. Adding *sex* improved model fit for the sanctuary model [*N* = 231 datapoints from females and 161 from males; *χ*^2^ = 6.72, df = 1, *P* = 0.01], showing that, across ages, *D*_*M*_ was lower in males than females. However, adding the interaction between *age* × *sex* did not further improve model fit [*χ*^2^ = 2.31, df = 1, *P* = 0.13], indicating that age-related increase in *D*_*M*_ was not different for males versus females. The results for the laboratory population paralleled the results from the sanctuary. There was also a positive age effect [*ß* = 0.24; *P* < 0.001]. Model fit was similarly improved with the inclusion of *sex* [*N* = 2015 datapoints from females and 1336 from males; *χ*^2^ = 4.41, df = 1, *P* = 0.04], with lower *D*_*M*_ in males, and inclusion of the *age × sex* interaction similarly did not improve fit [*χ*^2^ = 0.87, df = 1, *P* = 0.35]. For each sex, we compared the effect of age on *D*_*M*_ in sanctuary versus laboratory chimpanzees by extracting the standard effect coefficient for age in separate models for males and females at each facility (without controlling for potential *age* × *sex* interactions). While age slopes were similar for males of both groups, females at the laboratories exhibited steeper slopes compared to those at Ngamba (see Table 3 for age slopes from each model).

**Figure 3.**
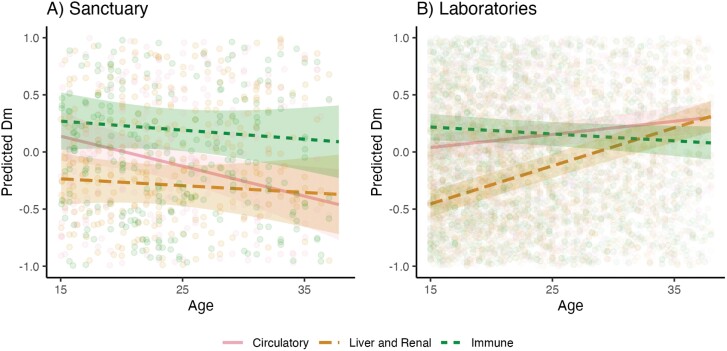
Age-related change in *D*_*M*_, split by *body system* at the (A) sanctuary and (B) laboratories. Predicted values are estimates of *D*_*M*_ (log-transformed and *z*-scored within each site) from models accounting for *subject identity, age*, *body system*, *age* × *body system* and *number of biomarkers* (and *site* for the laboratory model). Lines indicate linear fit; ribbons indicate 95% confidence intervals. Cardiometabolic biomarkers were removed from body system analysis due to low sample size. See Supplementary Fig. S5 for raw dysregulation scores, which increase across body systems at Ngamba

We finally explored whether dysregulation varied by body system, again testing this separately in the two populations (Supplementary Fig. S5; Fig. 3; Supplementary Table S5). We removed cardiometabolic biomarkers in these analyses, as they had a disproportionately low sample size compared to other systems across sites (see supplemental information). In the sanctuary base model, dysregulation did not appreciably change with age [*ß* = 0.01; *P* = 0.71]. Including *body system* did improve fit [*N* = 375 datapoints from the circulatory system, 384 from liver and renal, and 384 from immune; *χ*^2^ = 11.55, df = 2, *P* < 0.01]. Adding the interaction between *age* × *body system* did not further improve fit [*χ*^2^ = 3.76, df = 2, *P* = 0.15]: there was no difference in effect of *D*_*M*_ across body systems. We used the same approach for the laboratory data. Here, dysregulation increased with *age* in the base model for laboratory chimpanzees [ß = 0.09; *P* < 0.001]. Model fit was improved with the addition of both *body system* [*N* = 2725 datapoints from the circulatory system, 3104 from liver and renal, and 2292 from immune; *χ*^2^ = 50.71, df = 2, *p* < 0.001], as well as and the inclusion of an *age × body system* interaction [*χ*^2^ = 91.16, df = 2, *P* < 0.001]. Post-hoc tests showed that dysregulation in the laboratory population increased with age faster for circulatory than immune (*P* < 0.001) biomarkers; and faster for liver and renal than both immune (*P* < 0.001) and circulatory (*P* < 0.001) markers. For each body system, we compared the effect of age on *D*_*M*_ in sanctuary versus laboratory chimpanzees by extracting the standard effect coefficient for age in separate models for the circulatory, liver and renal, immune and cardiometabolic systems at each facility (without controlling for potential *age* × *body system* interactions). While we excluded cardiometabolic biomarkers in our main analysis due to unequal sample size, we were able to calculate age effects in system-specific models (though note the extremely large confidence intervals). The circulatory slope was steeper at the laboratories; the immune and cardiometabolic slopes were steeper at Ngamba; and the liver and renal slope was the same at both facilities (see Table 3 for age slopes from each model).

We conducted several additional analyses to check the robustness of these results. These yielded similar results as our primary facility analysis (see supplemental information for all details of these analyses). First, the analysis using the full age range from the laboratories confirmed our primary finding in showing higher dysregulation in the laboratory than the sanctuary, as well as steeper age-related change at the laboratories (with the same age-related slope for the laboratory sample as in the truncated dataset). The analyses using a pooled reference sample or only the common biomarkers across sites did not find a significant effect of facility, but both showed the same key interaction between age × facility, such that laboratory chimpanzees exhibited steeper increases in dysregulation with age compared to the sanctuary. Finally, we confirmed that our selection of a healthy (non-obese) reference sample was not too conservative by checking patterns using the upper quintile (top 20%) of sex-specific weight measurements as an overweight cutoff and found largely similar results to those reported in the main analyses.

## DISCUSSION

In this study, we asked whether physiological dysregulation, indexed by *Mahalanobis distance, D*_*M*_, differs based on living conditions in chimpanzees. We found that, relative to a within-group baseline, wild-born, semi-free-ranging chimpanzees exhibited overall lower dysregulation, and slower rates of age-associated increase, compared to captive chimpanzees living in laboratories. Our results extend prior findings of improved health among chimpanzees living in African sanctuaries compared with those in typical captive environments [12, 39, 40]. Chimpanzees in sanctuaries experience several notable lifestyle differences from conventional laboratory settings: large, forested areas that promote physical activity, diets comprised primarily of plant material, and large, social groups. Thus, our findings support an emerging literature that sedentary lifestyles and processed diets contribute to unhealthy aging patterns not only in humans but in our closest evolutionary relatives.

African sanctuaries are an interesting test case that allows us to separate the particular lifestyle factors that approximate the chimpanzees’ natural environment, such as diet composition and physical activity, from others that are more similar to captivity. Sanctuary chimpanzees receive routine medical care including preventative vaccination and treatment of illness and injury, mitigating risks of infectious disease likely to be experienced in the wild. For example, two recent studies revealed a vast majority of identified viruses in African sanctuary chimpanzees to be apathogenic [41, 42]. Furthermore, chimpanzees at African sanctuaries are buffered from the experience of resource shortages, unlike wild chimpanzees, as sanctuary-living chimpanzees receive some food provisioning in addition to the foods they can eat in their forest enclosures. Additionally, females are on hormonal contraception to prevent pregnancy [36], limiting potential influences of gestation and lactation on energetic costs and immune function. Thus, while we can determine that some features of the natural environment promote healthy aging in chimpanzees, our results do not allow us to extrapolate whether wild chimpanzees would experience faster or slower rates of physiological aging than the populations in the current study. While wild chimpanzees notably experience higher rates of mortality than captive chimpanzees across adulthood [52], rates of mortality in African sanctuary populations are generally very low [53]. We also caution that these different living contexts may be differently affected by survival bias and experience differing causes of mortality.

As in chimpanzees, human subsistence populations exhibit higher mortality but lower rates of degenerative disease than industrialized populations (i.e. improved cardiometabolic health, physical function, and cognitive health and slower epigenetic aging) [5, 6, 10, 54–56], supporting the hypothesis that sedentism and high-calorie diets are critical factors that affect healthy aging. However, in the one subsistence population for which it has been assessed, Tsimane forager-horticulturalists, physiological dysregulation proceeds slightly faster than in industrialized populations [9]. This highlights the need for additional comparative research across phylogeny and living conditions to parse the effect of specific lifestyle factors on primate aging.

Sanctuary chimpanzees in this study maintained a moderate rate of dysregulation relative to published scores for other primates, including humans. Age slopes at the laboratories (0.24) were similar to prior reports from captive chimpanzees (0.26) [24], while sanctuary slopes were lower (0.18). Slight differences in the slopes we obtained from the laboratory samples in the Primate Aging Database compared with this prior study were likely due to our selection of a different reference sample, one that is slightly older (15–20 years) to better represent the age at which growth is complete [52], and which excluded overweight individuals. Age slopes in sanctuary chimpanzees were only slightly higher than those of US humans (0.14) and the Tsimane (0.17) [9], consistent with chimpanzees’ moderately faster pace of life history compared to humans [25, 26]. However, it is important to note that one plausible explanation for this could be that our sample only included chimpanzees up to 38 years old. While this age range exceeds the median adult lifespan of chimpanzees, it does not represent the full lifespan experienced by this species, which can live past the age of 60 years [52]. Indeed, some human studies have documented exponential increases in dysregulation during old age [17, 19–21]. While we found no such exponential increase within the laboratory sample—which included older chimpanzees up to 58.25—this sample may be impacted by a survival bias, if only the healthiest individuals lived long enough to be present in the dataset at the oldest ages. We note that such a survivorship bias could not explain patterns in the sanctuary data, as none of the individuals in the sample died during this range.

We also identified a sex difference in dysregulation in both laboratory and sanctuary chimpanzees. Specifically, males had overall lower dysregulation scores than females in both populations, but their progression was no faster than females with aging. While a male–female health-survival paradox is robust for some human health indices [27], sex differences in physiological dysregulation are ambiguous in humans [28, 29] and other primates [24]. Our results are similar to those of Dansereau and colleagues, where dysregulation was overall higher in females, though one of two samples in their analysis also showed a faster age increase in dysregulation among males, which we did not detect in the current analyses. More broadly, though female chimpanzees show a reliable survival advantage [57, 58], evidence for a male health advantage is generally more equivocal [45, 59]. For instance, females in captivity exhibit a higher incidence of metabolic syndrome, including obesity and relatedly high blood pressure, whereas males are more often affected by myocardial fibrosis [51, 59].

Our system-by-system analyses also suggest that system-specific aging in chimpanzees differs based on living conditions. While dysregulation of the circulatory system progressed more rapidly in laboratory chimpanzees, mirroring patterns of global *D*_*M*_, dysregulation of the immune and cardiometabolic systems increased faster at the sanctuary. As mentioned above, both sanctuary and laboratory chimpanzees received veterinary care during the period of biomarker collection and, therefore, would have faced relatively low pathogen loads relative to wild adult chimpanzees. However, these groups may have experienced important differences in their *prior history* of infection. Most laboratory apes in this study were likely born on-site and, therefore, would have experienced similar environments throughout their lives (although individual housing history is unknown, and some laboratory chimpanzees born before 1973 could have been wild-caught) [60]. African sanctuary chimpanzees, on the other hand, are normally born into relatively high pathogen environments in the wild, and many are also victims of the bushmeat and pet trade before arrival at a sanctuary, where crude conditions could have exposed them to diseases. Other early influences of wild-born animals (e.g. energetic constraints during gestation and lactation) may also set the pace of aging. As early life adversity has long-lasting effects on the aging process across humans and other animals (e.g. on dysregulation of the HPA axis [61] and lifespan [62]), an important next step would be to examine how these earlier experiences influence aging.

One notable feature of our results is the apparently non-positive age slopes observed for some body systems. While neutral slopes have been observed in system-by-system studies of humans, as well (e.g. lipids [23]), this is nonetheless a surprising result. One possibility is that this stems from within-individual shifts in dysregulation scores that were influenced by lifestyle interventions. Unlike in the wild, captive apes are fed controlled diets, which are increasingly regulated to manage their weight and associated cardiovascular health in response to indications that these chimpanzees have emerging health problems [36, 63, 64]. This is not dissimilar to diet and exercise interventions implemented in industrialized human populations. Along these lines, total cholesterol declined with age in one of the laboratories examined in this study, suggesting that at least by some metrics, they may be getting healthier over time [12]. While it is not possible for us to directly assess why some metrics of health may improve with age, it does raise the possibility that diet or lifestyle interventions can alter trajectories of physiological dysregulation in primates.

Finally, because lifestyle affected not just the magnitude of dysregulation, but the importance of particular systems in driving global dysregulation, this does suggest that dysregulation scores are not completely insensitive to biomarker selection. In smaller biomarker datasets, it is important to investigate the possibility of biases in biomarker composition between samples. For example, higher dysregulation of the immune and cardiometabolic systems at Ngamba could reflect the inclusion of unique targeted biomarkers (e.g. measures of inflammation and body fat that are not present in the Primate Aging Database). However, our global results were robust to the use of a smaller set of biomarkers common to each population, and we also found that no one biomarker disproportionately contributed to our results. As such, our overall results are consistent with the complex systems theory of aging, in which integrated, resilient networks explain organismal aging more powerfully than do individual biomarkers [14–16].

## CONCLUSIONS AND IMPLICATIONS

Age-related increases of physiological dysregulation in both chimpanzee groups were more similar to current data on humans than to those from other primate species [9, 24]—that is, lower and less variable. This suggests that within-species constraints of *D*_*M*_ may extend beyond humans and more generally accords with prior findings that aging parameters are phylogenetically conserved [11, 65]. Concurrently, sanctuary chimpanzees showed slower global rates of aging than laboratory chimpanzees, despite experiencing faster dysregulation of several body systems. This supports the idea that lifestyle effects on the human healthspan [2, 6] are conserved from deeper in our evolutionary history. It is also consistent with accumulating evidence that animals can show remarkable resilience to early life adversity [66–68]. On the whole, these findings bolster semi-free-ranging chimpanzees as a valuable evolutionarily model for healthy human aging.

## Supplementary Material

eoae010_suppl_Supplementary_Data_S1

eoae010_suppl_Supplementary_Data_S2

eoae010_suppl_Supplementary_Data_S3

eoae010_suppl_Supplementary_Data_S4

eoae010_suppl_Supplementary_Material

## References

[1] Lieberman DE. The Story of the Human Body: Evolution, Health, and Disease. NY: Pantheon Books, 2013. 27875612

[2] Yusuf S , Joseph P, Rangarajan S et al. Modifiable risk factors, cardiovascular disease, and mortality in 155 722 individuals from 21 high-income, middle-income, and low-income countries (PURE): a prospective cohort study. Lancet 2020;395:795–808. 31492503 10.1016/S0140-6736(19)32008-2 PMC8006904

[3] Shammas MA. Telomeres, lifestyle, cancer, and aging. Curr Opin Clin Nutr Metab Care 2011;14:28–34. 21102320 10.1097/MCO.0b013e32834121b1 PMC3370421

[4] Sharifi-Rad M , Anil Kumar NV, Zucca P et al. Lifestyle, oxidative stress, and antioxidants: back and forth in the pathophysiology of chronic diseases. Front Physiol 2020;11:694. 32714204 10.3389/fphys.2020.00694 PMC7347016

[5] Kaplan H , Thompson RC, Trumble BC et al. Coronary atherosclerosis in indigenous South American Tsimane: a cross-sectional cohort study. The Lancet 2017;389:1730–9. 10.1016/S0140-6736(17)30752-3 PMC6028773 28320601

[6] Pontzer H , Wood BM, Raichlen DA. Hunter-gatherers as models in public health. Obes Rev 2018;19:24–35. 30511505 10.1111/obr.12785

[7] Gurven M , Gomes CM. Mortality, senescence, and life span. In: Muller MN, Wrangham RW, Pilbeam DR (eds.). Chimpanzees and Human Evolution. Cambridge, MA: Belknap Press of Harvard University Press, 2017, 181–216.

[8] Gurven M , Trumble BC, Stieglitz J et al. Cardiovascular disease and type 2 diabetes in evolutionary perspective: a critical role for helminths? Evol Med Public Health 2016;1:338–57. 10.1093/emph/eow028 PMC5101910 27666719

[9] Kraft TS , Stieglitz J, Trumble BC et al. Multi-system physiological dysregulation and ageing in a subsistence population. Philos Trans R Soc B Biol Sci 2020;375:20190610. 10.1098/rstb.2019.0610 PMC7540955 32951553

[10] Horvath S , Gurven M, Levine ME et al. An epigenetic clock analysis of race/ethnicity, sex, and coronary heart disease. Genome Biol 2016;17:171. 27511193 10.1186/s13059-016-1030-0 PMC4980791

[11] Colchero F , Aburto JM, Archie EA et al. The long lives of primates and the ‘invariant rate of ageing’ hypothesis. Nat Commun 2021;12:3666. 34135334 10.1038/s41467-021-23894-3 PMC8209124

[12] Cole MF , Cantwell A, Rukundo J et al. Healthy cardiovascular biomarkers across the lifespan in wild-born chimpanzees (*Pan troglodytes*). Philos Trans R Soc B: Biol Sci 2020;375:20190609. 10.1098/rstb.2019.0609 PMC7540951 32951545

[13] Ely JJ , Zavaskis T, Lammey ML. Censored data analysis reveals effects of age and hepatitis C infection on C-reactive protein levels in healthy adult chimpanzees (*Pan troglodytes*). J Biomark 2013;2013:1–13. 10.1155/2013/709740 PMC4437358 26317021

[14] Cohen AA , Ferrucci L, Fülöp T et al. A complex systems approach to aging biology. Nat Aging 2022;2:580–91. 37117782 10.1038/s43587-022-00252-6 PMC12007111

[15] Kirkwood TBL. Understanding the odd science of aging. Cell 2005;120:437–47. 15734677 10.1016/j.cell.2005.01.027

[16] Weinert BT , Timiras PS. Invited review: theories of aging. J Appl Physiol 2003;95:1706–16. 12970376 10.1152/japplphysiol.00288.2003

[17] Cohen AA , Milot E, Yong J et al. A novel statistical approach shows evidence for multi-system physiological dysregulation during aging. Mech Ageing Dev 2013;134:110–7. 23376244 10.1016/j.mad.2013.01.004 PMC3971434

[18] De Maesschalck R , Jouan-Rimbaud D, Massart DL. The Mahalanobis distance. Chemom Intell Lab Syst 2000;50:1–18.

[19] Cohen AA , Milot E, Li Q et al. Cross-population validation of statistical distance as a measure of physiological dysregulation during aging. Exp Gerontol 2014;57:203–10. 24802990 10.1016/j.exger.2014.04.016 PMC4428144

[20] Lu Y , Gwee X, Chua DQL et al. Physiological dysregulation, frailty, and impacts on adverse health and functional outcomes. Front Med 2021;8:751022. 10.3389/fmed.2021.751022 PMC8569246 34746185

[21] Milot E , Morissette-Thomas V, Li Q et al. Trajectories of physiological dysregulation predicts mortality and health outcomes in a consistent manner across three populations. Mech Ageing Dev 2014;141-142:56–63. 25454986 10.1016/j.mad.2014.10.001 PMC4310774

[22] Cohen AA , Li Q, Milot E et al. Statistical distance as a measure of physiological dysregulation is largely robust to variation in its biomarker composition. Koomen JM (ed.). PLoS One 2015;10:e0122541. 25875923 10.1371/journal.pone.0122541 PMC4395377

[23] Li Q , Wang S, Milot E et al. Homeostatic dysregulation proceeds in parallel in multiple physiological systems. Aging Cell 2015;14:1103–12. 26416593 10.1111/acel.12402 PMC4693454

[24] Dansereau G , Wey TW, Legault V et al. Conservation of physiological dysregulation signatures of aging across primates. Aging Cell 2019;18:e12925. 30746836 10.1111/acel.12925 PMC6413749

[25] Charnov EL. Evolution of life history variation among female mammals. Proc Natl Acad Sci USA 1991;88:1134–7. 1996315 10.1073/pnas.88.4.1134 PMC50971

[26] Kaplan H , Hill K, Lancaster J et al. A theory of human life history evolution: diet, intelligence, and longevity. Evol Anthropol: Issues, News, Rev 2000;9:156–85.

[27] Lindahl-Jacobsen R , Hanson HA, Oksuzyan A et al. The male-female health-survival paradox and sex differences in cohort life expectancy in Utah, Denmark, and Sweden 1850-1910. Ann Epidemiol 2013;23:161–6. 23453386 10.1016/j.annepidem.2013.02.001 PMC3651922

[28] Arbeev KG , Cohen AA, Arbeeva LS et al. Optimal versus realized trajectories of physiological dysregulation in aging and their relation to sex-specific mortality risk. Front Public Health 2016;4:1–12. DOI: 10.3389/fpubh.2016.00003 26835445 PMC4725219

[29] Cohen AA , Legault V, Li Q et al. Men sustain higher dysregulation levels than women without becoming frail. J Gerontol Ser A 2018;73:175–84. 10.1093/gerona/glx146 PMC5861919 28977345

[30] Fowler MA , Paquet M, Legault V et al. Physiological predictors of reproductive performance in the European Starling (*Sturnus vulgaris*). Front Zool 2018;15:45. 30479645 10.1186/s12983-018-0288-3 PMC6249724

[31] Milot E , Cohen AA, Vézina F et al. A novel integrative method for measuring body condition in ecological studies based on physiological dysregulation. Freckleton R (ed.). Methods Ecol Evol 2014;5:146–55.

[32] Gurven M , Lieberman DE. WEIRD bodies: mismatch, medicine and missing diversity. Evol Hum Behav 2020;41:330–40. 33100820 10.1016/j.evolhumbehav.2020.04.001 PMC7584376

[33] Gurven M , Trumble BC, Stieglitz J et al. High resting metabolic rate among Amazonian forager-horticulturalists experiencing high pathogen burden. Am J Phys Anthropol 2016;161:414–25. 27375044 10.1002/ajpa.23040 PMC5075257

[34] Emery Thompson M , Rosati AG, Snyder-Mackler N. Insights from evolutionarily relevant models for human ageing. Philos Trans R Soc B Biol Sci 2020;375:20190605. 10.1098/rstb.2019.0605 PMC7540954 32951550

[35] Muller MN , Wrangham RW, Pilbeam D eds. Chimpanzees and Human Evolution. Boston, MA: Belknap Press of Harvard University Press, 2017.

[36] Stokes R , Tully G, Rosati AG. Pan African sanctuary alliance: securing a future for the African great apes. Int Zoo Yearb 2018;52:173–81.

[37] Rosati AG , Sabbi KH, Bryer MAH et al. Observational approaches to chimpanzee behavior in an African sanctuary: implications for research, welfare, and capacity‐building. Am J Primatol 2023;85:e23534. 37461356 10.1002/ajp.23534 PMC10530331

[38] Wobber V , Hare B. Psychological health of orphan bonobos and chimpanzees in African sanctuaries. PLoS One 2011;6:e17147. 21666743 10.1371/journal.pone.0017147 PMC3110182

[39] Atencia R , Stöhr EJ, Drane AL et al. Heart rate and indirect blood pressure responses to four different field anesthetic protocols in wild-born captive chimpanzees (*Pan troglodytes*). J Zoo Wildl Med 2017;48:636–44. 28920777 10.1638/2016-0181.1

[40] Curry BA , Drane AL, Atencia R et al. Body mass and growth rates in captive chimpanzees (*Pan troglodytes*) cared for in African wildlife sanctuaries, zoological institutions, and research facilities. Zoo Biol 2023;42:98–106. 35815730 10.1002/zoo.21718 PMC10084351

[41] Dunay E , Owens LA, Dunn CD et al. Viruses in sanctuary chimpanzees across Africa. Am J Primatol 2023;85:1–16. DOI: 10.1002/ajp.23452. PMC9812903 36329642

[42] Dunay E , Rukundo J, Atencia R et al. Viruses in saliva from sanctuary chimpanzees (*Pan troglodytes*) in Republic of Congo and Uganda. Berber E (ed.). PLoS One 2023;18:e0288007. 37384730 10.1371/journal.pone.0288007 PMC10310015

[43] Rosati AG , Emery Thompson M, Atencia R et al. Distinct developmental trajectories for risky and impulsive decision-making in chimpanzees. J Exp Psychol Gen 2023;152:1551–64. DOI: 10.1037/xge0001347. 36689365 PMC10271938

[44] Muller MN , Enigk DK, Fox SA et al. Aggression, glucocorticoids, and the chronic costs of status competition for wild male chimpanzees. Horm Behav 2021;130:104965. 33676127 10.1016/j.yhbeh.2021.104965 PMC8043126

[45] Negrey JD , Reddy RB, Scully EJ et al. Simultaneous outbreaks of respiratory disease in wild chimpanzees caused by distinct viruses of human origin. Emerging Microb Infect 2019;8:139–49. 10.1080/22221751.2018.1563456 PMC6455141 30866768

[46] Wobber V , Wrangham R, Hare B. Bonobos exhibit delayed development of social behavior and cognition relative to chimpanzees. Curr Biol 2010;20:226–30. 20116251 10.1016/j.cub.2009.11.070

[47] González NT , Otali E, Machanda Z et al. Urinary markers of oxidative stress respond to infection and late-life in wild chimpanzees. Serrano E (ed.). PLoS One 2020;15:e0238066. 32916689 10.1371/journal.pone.0238066 PMC7486137

[48] Fulk, R, M. Loomis, C. Garland. Nutrition of captive chimpanzees. The Care and Management of Chimpanzees in Captive Environments. Chimpanzee Species Survival Plan - Husbandry Manual. American Association of Zoos and Aquariums, 1992.

[49] Hill K , Boesch C, Goodall J et al. Mortality rates among wild chimpanzees. J Hum Evol 2001;40:437–50. 11322804 10.1006/jhev.2001.0469

[50] Ely JJ , Zavaskis T, Lammey ML. Hypertension increases with aging and obesity in chimpanzees (*Pan troglodytes*): hypertension in chimpanzees. Zoo Biol 2013;32:79–87. 22968757 10.1002/zoo.21044 PMC3537917

[51] Videan EN , Fritz J, Murphy J. Development of guidelines for assessing obesity in captive chimpanzees (*Pan troglodytes*). Zoo Biol 2007;26:93–104. 19360564 10.1002/zoo.20122

[52] Emery Thompson M , Sabbi K. Evolutionary demography of the great apes. In: Burger O, Lee R, Sear R (eds.). Human Evolutionary Demography. Cambridge, UK: Open Book Publishers, 2021. DOI: 10.11647/OBP.0251.

[53] Faust LJ , Cress D, Farmer KH et al. Predicting capacity demand on sanctuaries for African chimpanzees (*Pan troglodytes)*. Int J Primatol 2011;32:849–64.

[54] Gatz M , Mack WJ, Chui HC et al. Prevalence of dementia and mild cognitive impairment in indigenous Bolivian forager‐horticulturalists. Alzheimers Dement 2023;19:44–55. 35262289 10.1002/alz.12626 PMC9458772

[55] Raichlen DA , Pontzer H, Harris JA et al. Physical activity patterns and biomarkers of cardiovascular disease risk in hunter-gatherers. Am J Hum Biol 2017;29:1–13. 10.1002/ajhb.22919 27723159

[56] Sayre MK , Pontzer H, Alexander GE et al. Ageing and physical function in East African foragers and pastoralists. Philos Trans R Soc B: BiolSci 2020;375:20190608. 10.1098/rstb.2019.0608 PMC7540945 32951542

[57] Muller MN , Wrangham RW. Mortality rates among Kanyawara chimpanzees. J Hum Evol 2014;66:107–14. 24374229 10.1016/j.jhevol.2013.10.004

[58] Wood BM , Watts DP, Mitani JC et al. Favorable ecological circumstances promote life expectancy in chimpanzees similar to that of human hunter-gatherers. J Hum Evol 2017;105:41–56. 28366199 10.1016/j.jhevol.2017.01.003 PMC5526083

[59] Lowenstine LJ , McManamon R, Terio KA. Comparative pathology of aging great apes: Bonobos, chimpanzees, gorillas, and orangutans. Vet Pathol 2016;53:250–76. 26721908 10.1177/0300985815612154

[60] Weiss A , King JE, Hopkins WD. A cross-setting study of chimpanzee (*Pan troglodytes*) personality structure and development: zoological parks and Yerkes National Primate Research Center. Am J Primatol 2007;69:1264–77. 17397036 10.1002/ajp.20428 PMC2654334

[61] Bunea IM , Szentágotai-Tătar A, Miu AC. Early-life adversity and cortisol response to social stress: a meta-analysis. Transl Psychiatry 2017;7:1274. 29225338 10.1038/s41398-017-0032-3 PMC5802499

[62] Tung J , Archie EA, Altmann J et al. Cumulative early life adversity predicts longevity in wild baboons. Nat Commun 2016;7:11181. 27091302 10.1038/ncomms11181 PMC4838827

[63] Varki N , Anderson D, Herndon JG et al. Original Article: heart disease is common in humans and chimpanzees, but is caused by different pathological processes. Evol Appl 2009;2:101–12. 25567850 10.1111/j.1752-4571.2008.00064.x PMC3352420

[64] Clay AW , Crane MM, Bloomsmith MA. Weight management towards physiological and behavioral wellbeing for chimpanzees living under human care. Zoo Biol 2022;41:200–17. 35037298 10.1002/zoo.21668

[65] Bronikowski AM , Altmann J, Brockman DK et al. Aging in the natural world: comparative data reveal similar mortality patterns across primates. Science 2011;331:1325–8. 21393544 10.1126/science.1201571 PMC3396421

[66] Daskalakis NP , Bagot RC, Parker KJ et al. The three-hit concept of vulnerability and resilience: toward understanding adaptation to early-life adversity outcome. Psychoneuroendocrinology 2013;38:1858–73. 23838101 10.1016/j.psyneuen.2013.06.008 PMC3773020

[67] Morrison RE , Eckardt W, Stoinski TS et al. Cumulative early-life adversity does not predict reduced adult longevity in wild gorillas. Curr Biol 2023;33:2307–14.e4. 37192615 10.1016/j.cub.2023.04.051 PMC10264970

[68] Stevens HE , Leckman JF, Coplan JD et al. Risk and resilience: early manipulation of macaque social experience and persistent behavioral and neurophysiological outcomes. J Am Acad Child Adolesc Psychiatry 2009;48:114–27. 19127170 10.1097/CHI.0b013e318193064c

