# Supplementary material for: Age-related physiological dysregulation progresses slowly in semi-free-ranging chimpanzees
Source: Evol Med Public Health. 2024 Jun 19;12(1):129–42. doi: 10.1093/emph/eoae010 (PMC11375048; doi:10.1093/emph/eoae010)
Supplement: eoae010_suppl_Supplementary_Material [file eoae010_suppl_supplementary_material.docx]

**SUPPLEMENTAL INFORMATION**

Age-related physiological dysregulation progresses slowly in semi-free-ranging chimpanzees

Megan F. Cole, Paige Barnes, Isabelle G. Monroe, Joshua Rukundo, Melissa Emery Thompson,

& Alexandra G. Rosati

**1. Supplemental methods**

***Biomarker selection***

From complete blood count tests, we included only biomarkers which were independent and clinically relevant following the approach of prior work [1,2]. For example, for anything we included as a count (red and white blood cell counts) we excluded corresponding percentages (red and white blood cell composition). Because some measures were ratios, we included one but not both component measures. For the ratio of protein measures albumin to globulin (ALB/GLOB), we included the corresponding denominator GLOB, but not the uncorrected numerator ALB. We also included the ratio of ALB to total protein count (ALB/TP), but not TP alone. For the total cholesterol to high-density lipoprotein ration (TC/HDL), we included TC but not HDL because it was uniquely available across all four sites. We finally excluded immature granulocyte count (IG) because it is optimal at 0, but 0 values would appear highly dysregulated after z-scoring.

We also removed extreme outliers for each biomarker. This included biologically implausible values (based on human references); values that were vastly incongruent with related measures; and extreme statistical outliers (e.g., values that were twice as high as the next highest observation). Such outliers likely reflect typos or very poor health, both of which would bias our investigation of healthy aging.

***Data transformation***

As noted in the main text, we checked all individual biomarkers’ distribution at each site, and then log- or square-root-transformed them as relevant for the reference sample to approach normality. Tables S1-S2 list how individual biomarkers were transformed at each site, and their correlations to age and *D_M_*. Correlation matrices among available biomarkers are located in separate supplemental excel files.

**Table S1. Biomarkers included in *D_M_* calculation, by body system and site.** Data transformations are indicated as relevant. *Measured for only two years at Ngamba

|  | **Ngamba** | **Alamogordo** | | **Yerkes** | **MD Anderson** |
| --- | --- | --- | --- | --- | --- |
| *Circulatory system* | | | | | |
| RBC | Untransformed | | Untransformed | Untransformed | Untransformed |
| HGB | Untransformed | | Untransformed | Untransformed | Untransformed |
| %HCT | Untransformed | | Untransformed | Untransformed | Untransformed |
| MCV | Untransformed | | Untransformed | Untransformed | Untransformed |
| MCH | Untransformed | | Untransformed | Untransformed | Untransformed |
| MCHC | Untransformed | | Untransformed | Untransformed | Untransformed |
| PLT | Square root | | Square root | Square root | Square root |
| %RDW | Log | |  |  |  |
| MPV | Untransformed | |  |  |  |
| %PCT | Log | |  |  |  |
| %PDW | Log | |  |  |  |
| %P-LCR | Square root | |  |  |  |
| FE |  | | Square root |  |  |
| *Liver and renal system* | | | | | |
| CR | Log | | Log | Log | Log |
| GLOB | Untransformed | | Untransformed | Untransformed | Square root |
| ALP | Square root | | Square root | Square root | Square root |
| NA | Untransformed | | Untransformed | Untransformed | Untransformed |
| K | Log | | Untransformed | Untransformed | Untransformed |
| BUN/CR | Log | | Log | Log | Log |
| ALB/GLOB | Untransformed | | Untransformed | Untransformed | Log |
| ALB/TP | Untransformed | | Untransformed | Untransformed | Untransformed |
| TBIL | Untransformed | | Square root | Square root | Square root |
| GGT | Log | | Log |  | Log |
| CL | Untransformed | | Square root |  |  |
| HCO3 | Untransformed | | Square root |  |  |
| AST | Log | |  |  |  |
| ALT | Log | |  |  |  |
| DBIL | Untransformed | |  |  |  |
| IBIL | Square root | |  |  |  |
| CA |  | | Untransformed | Untransformed | Untransformed |
| CPK |  | | Log | Log | Square root |
| LDH |  | | Square root | Square root | Square root |
| SGPT |  | | Square root | Square root | Square root |
| SGOT |  | | Log | Log | Log |
| PO4 |  | | Log | Untransformed | Untransformed |
| UA |  | | Untransformed |  | Log |
| MG |  | | Untransformed |  |  |
| AMYL |  | | Square root |  |  |
| LIPA |  | | Square root |  |  |
| *Immune system* | | | | | |
| WBC | Log | | Square root | Square root | Log |
| LYM | Log | | Square root | Log | Untransformed |
| MON | Log | | Log | Log | Log |
| EOS | Square root | | Log | Log | Log |
| BAS | Log | | Untransformed | Log | Log |
| CRP | Log* | |  |  |  |
| IL6 | Square root* | |  |  |  |
| OHdG | Square root* | |  |  |  |
| Isoprostanes | Untransformed* | |  |  |  |
| TAC | Square root* | |  |  |  |
| Neopterin | Log* | |  |  |  |
| NEU | Log | |  |  |  |
| NEU-B |  | | Log | Log | Log |
| NEU-S |  | | Log | Square root | Square root |
| *Cardiometabolic system* | | | | | |
| Body weight | Square root | | Square root | Square root | Square root |
| GLU | Untransformed | | Untransformed | Untransformed | Untransformed |
| TC | Square root* | | Square root | Square root | Square root |
| TRG | Square root* | | Square root | Square root | Square root |
| TC/HDL | Untransformed* | |  |  | Untransformed |
| Inguinal skinfold | Untransformed* | |  |  |  |
| Biceps skinfold | Untransformed* | |  |  |  |
| Triceps skinfold | Untransformed* | |  |  |  |
| Subscapular skinfold | Untransformed* | |  |  |  |
| Upper arm | Untransformed* | |  |  |  |

**Table S2. Correlations of individual biomarkers to age and *D_M_*, by body system and site.** Bolded correlations are significantly different from zero.

|  | **Ngamba** | | **Alamogordo** | | **Yerkes** | | **MD Anderson** | |
| --- | --- | --- | --- | --- | --- | --- | --- | --- |
|  | **Age** | ***D_M_*** | **Age** | ***D_M_*** | **Age** | ***D_M_*** | **Age** | ***D_M_*** |
| *Circulatory system* | | | | | | | | |
| RBC | **-0.27** | **-0.27** | **0.07** | **-0.13** | **-0.22** | **-0.22** | -0.02 | 0.06 |
| HGB | **-0.25** | **-0.19** | **0.07** | **0.36** | **-0.22** | **-0.30** | **-0.12** | **0.08** |
| %HCT | **-0.21** | **-0.21** | **0.10** | **-0.15** | **-0.19** | **-0.29** | **-0.15** | -0.02 |
| MCV | **0.11** | 0.01 | **0.07** | **-0.19** | **0.08** | **-0.08** | **-0.13** | 0.07 |
| MCH | 0.04 | **0.16** | 0.01 | **0.11** | -0.01 | **-0.12** | **-0.10** | 0.04 |
| MCHC | -0.07 | **0.18** | -0.06 | 0.06 | **-0.13** | **-0.09** | **-0.10** | 0.01 |
| PLT | **-0.17** | **-0.23** | **-0.11** | **0.09** | 0.00 | 0.02 | -0.07 | -0.05 |
| %RDW | -0.09 | 0.03 |  |  |  |  |  |  |
| MPV | **0.15** | **-0.11** |  |  |  |  |  |  |
| %PCT | -0.10 | **-0.26** |  |  |  |  |  |  |
| %PDW | 0.10 | 0.05 |  |  |  |  |  |  |
| %P-LCR | 0.04 | 0.05 |  |  |  |  |  |  |
| FE |  |  | -0.07 | 0.00 |  |  |  |  |
| *Liver and renal system* | | | | | | | | |
| CR | 0.01 | **0.12** | 0.02 | **0.09** | **-0.07** | **-0.08** | -0.06 | **-0.12** |
| GLOB | **0.26** | 0.12 | **0.22** | **0.07** | **0.19** | **0.38** | **0.07** | **0.17** |
| ALP | **-0.10** | -0.06 | **0.12** | 0.05 | -0.05 | **0.13** | **-0.18** | **0.10** |
| NA | 0.05 | -0.02 | **-0.07** | **-0.09** | 0.03 | -0.04 | **0.15** | **-0.24** |
| K | **-0.24** | 0.00 | **-0.09** | **-0.09** | **-0.37** | -0.03 | **-0.37** | **-0.13** |
| BUN/CR | 0.00 | **0.15** | **-0.07** | **-0.15** | **0.18** | **0.15** | **-0.11** | **0.08** |
| ALB/GLOB | **-0.21** | **-0.20** | **-0.19** | **-0.17** | **-0.35** | **-0.41** | **-0.11** | **-0.16** |
| ALB/TP | **-0.15** | **-0.19** | **-0.18** | **-0.20** | **-0.33** | **-0.45** | -0.06 | -0.01 |
| TBIL | 0.06 | -0.07 | **0.11** | 0.05 | -0.06 | **-0.09** | -0.03 | **0.10** |
| GGT | 0.00 | 0.04 | **0.24** | **0.31** |  |  | -0.06 | **0.34** |
| CL | **0.14** | **-0.19** | -0.02 | **-0.33** |  |  |  |  |
| HCO3 | -0.05 | **0.19** | 0.05 | -0.02 |  |  |  |  |
| AST | **0.21** | 0.08 |  |  |  |  |  |  |
| ALT | **0.15** | 0.00 |  |  |  |  |  |  |
| DBIL | 0.06 | -0.08 |  |  |  |  |  |  |
| IBIL | 0.04 | -0.06 |  |  |  |  |  |  |
| CA |  |  | -0.04 | **0.14** | **-0.16** | **0.07** | -0.07 | **0.19** |
| CPK |  |  | 0.07 | -0.03 | **-0.13** | -0.04 | **-0.21** | **0.24** |
| LDH |  |  | **0.09** | -0.05 | **-0.15** | **-0.12** | -0.11 | **0.17** |
| SGPT |  |  | -0.03 | -0.04 | **-0.08** | **-0.12** | 0.03 | **0.14** |
| SGOT |  |  | **0.09** | 0.01 | 0.00 | -0.01 | **-0.11** | **0.17** |
| PO4 |  |  | **-0.21** | **-0.10** | -0.04 | **0.07** | -0.08 | **0.15** |
| UA |  |  | **0.18** | **0.23** |  |  | 0.05 | **0.26** |
| MG |  |  | 0.08 | -0.02 |  |  |  |  |
| AMYL |  |  | **-0.11** | -0.06 |  |  |  |  |
| LIPA |  |  | **0.17** | 0.14 |  |  |  |  |
| *Immune system* | | | | | | | | |
| WBC | **-0.13** | 0.01 | 0.04 | **0.11** | 0.01 | 0.05 | **-0.11** | 0.06 |
| LYM | **-0.37** | -0.06 | -0.04 | 0.04 | **0.25** | -0.03 | **-0.23** | -0.05 |
| MON | -0.04 | 0.04 | 0.02 | **0.17** | **0.07** | **0.09** | **0.10** | 0.05 |
| EOS | 0.04 | -0.01 | -0.03 | 0.07 | 0.02 | **-0.09** | **-0.07** | -0.04 |
| BAS | **-0.11** | 0.07 | -0.03 | 0.01 | 0.08 | **-0.39** | 0.04 | **0.06** |
| IG | 0.01 | -0.11 |  |  |  |  |  |  |
| CRP | 0.15 | 0.06 |  |  |  |  |  |  |
| IL6 | -0.08 | -0.16 |  |  |  |  |  |  |
| OHdG | -0.10 | 0.09 |  |  |  |  |  |  |
| Isoprostanes | 0.07 | 0.22 |  |  |  |  |  |  |
| TAC | 0.12 | 0.11 |  |  |  |  |  |  |
| Neopterin | 0.02 | -0.05 |  |  |  |  |  |  |
| NEU | 0.02 | -0.05 |  |  |  |  |  |  |
| NEU-B |  |  | -0.05 | **0.11** |  |  | -0.03 | **0.09** |
| NEU-S |  |  | 0.01 | **-0.14** | **-0.24** | -0.01 | **0.15** | 0.04 |
| *Cardiometabolic system* | | | | | | | | |
| Body weight | **0.14** | 0.00 | 0.03 | **0.25** | -0.09 | 0.09 | **0.19** | **0.32** |
| GLU | 0.19 | **0.31** | **0.16** | **0.07** | **0.13** | 0.00 | 0.05 | **0.13** |
| TC | 0.14 | 0.21 | **0.08** | **0.14** | **0.21** | **0.12** | **-0.18** | **0.26** |
| TRG | -0.20 | -0.06 | 0.06 | **0.22** | **0.18** | **0.41** | 0.08 | **0.29** |
| TC/HDL | 0.14 | 0.18 |  |  |  |  | 0.07 | **0.58** |
| Inguinal skinfold | **0.44** | **0.39** |  |  |  |  |  |  |
| Biceps skinfold | **0.44** | 0.18 |  |  |  |  |  |  |
| Triceps skinfold | **0.28** | **0.28** |  |  |  |  |  |  |
| Subscapular skinfold | 0.06 | 0.06 |  |  |  |  |  |  |
| Upper arm | -0.06 | 0.05 |  |  |  |  |  |  |

***Weight cutoff for healthy reference samples***

As described in the main text, we created site-specific reference samples from non-overweight individuals aged 15-20 years. We used the overweight threshold originally proposed in Videan et al. [3], which here corresponded to a natural break in female weight of 54.4 kg in the combined dataset (across the sanctuary and all three laboratory sites). There was an analogous break in the male weight distribution at 67.4 kg (Figure S2). 96% of observations (109/114) from young adults at Ngamba were at a healthy weight by this standard and therefore included in the reference sample, while only 37% (477/1,286) were at the laboratories. See below for use of a less conservative overweight cutoff, which produced very similar results.

**
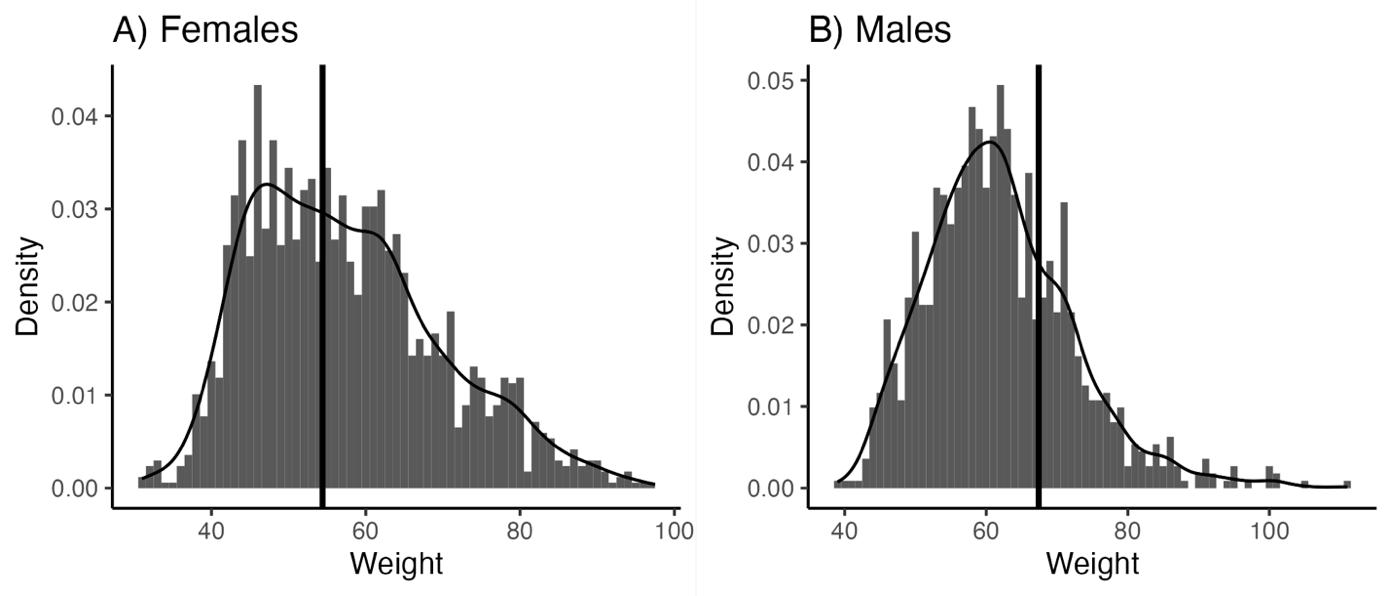
**

**Figure S1. Weight (kg) distributions for A) females and B) males.** Vertical lines indicate overweight cutoff: 54.4 kg and 67.4 kg for females and males, respectively.

***Exclusion of Ngamba 2011 data***

As noted in the main manuscript, our analyses comprised 11 years of data from the sanctuary site spanning 2012-2022. There was one additional year of data collected during 2011, but visualization of the data revealed unusually high dysregulation scores in this first year (Figure S2). Note that higher scores in 2016 and 2017 likely reflect the inclusion of additional, unique biomarkers due to intensive sampling for specific research projects during those years (Table 2), and the inclusion of more biomarkers generally increases Dm scores as discussed in the main manuscript. Unlike in later years, samples from 2011 were also analyzed at two different local laboratories, including one that assayed samples only in that year and may have used different assay references. While the overall results reported in the manuscript (e.g., that the sanctuary had lower dysregulation and less age-related increases in dysregulation than laboratories) held with the inclusion of this data, high values in the first year of sampling skewed results toward predicting negative age trajectories at Ngamba. Given the biological implausibility of this finding, as well as the discrepancy in how assays were conducted in 2011, we removed this year from the final dataset.

***
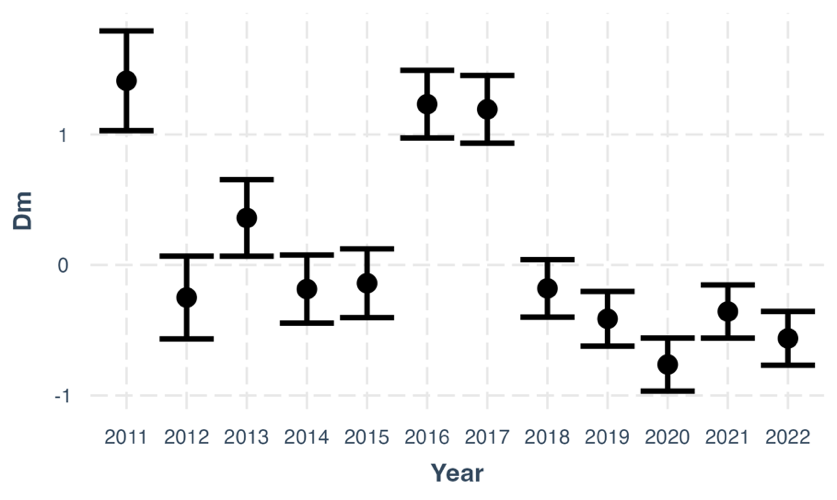
***

**Figure S2. *D_M_* scores at Ngamba for all available years.** *D_M_* is log transformed and z-scored to one standard deviation. 2011 was removed from analysis 1) because complete blood count tests were conducted at two separate laboratories that may have used non-comparable reference ranges; and 2) for being an extreme outlier compared to subsequent years. High dysregulation in 2016 and 2017 was likely driven by the large number of additional biomarkers collected during those years.

**2. Main analyses**

***Comparison of sanctuary and laboratory chimpanzees***

The primary dataset comprised 3,743 datapoints, 392 from Ngamba and 3,351 from the laboratories. Raw *D_M_* scores are shown in Figure S3, where laboratory and sanctuary chimpanzees follow a similar age-related increase. Model-predicted values in Figure 1 show that dysregulation progresses faster in laboratory chimpanzees after accounting for relevant covariates. Table S3 compares model effects of the main analysis with supplemental analyses using the full age range at laboratories; a pooled reference sample; and 25 common biomarkers available at all four sites.


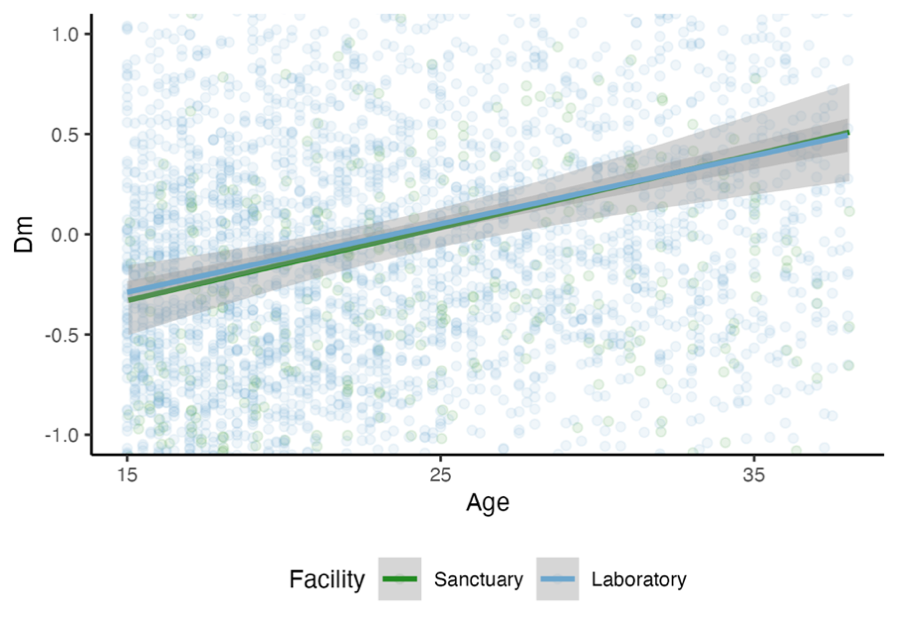


**Figure S3. Age-related change in *D_M_*, split by *facility*.** *D_M_* is log transformed and z-scored to one standard deviation. Lines indicate linear fit; ribbons indicate 95% confidence intervals.

**Table S3. Effects of facility models.** All analyses use an age-matched sample (15-38 years), all available biomarkers, and site-specific reference samples, unless otherwise noted. The full laboratory age range incudes all available ages at the laboratories (up to age 58.28). The pooled reference analysis combines all available observations of healthy young adults at all sites. The common biomarker analysis uses only the 25 biomarkers available across all four sites. The alternative overweight cutoff uses a more conservative threshold for the healthy reference sample. Parameters are from full models.

|  | **Main analysis** | **Full laboratory age range** | **Pooled reference** | **Common biomarkers** | **Alternative overweight cutoff** |
| --- | --- | --- | --- | --- | --- |
| Age  *(standardized)* | *Est*. = 0.03  *SE =* 0.06  *t =* 0.41  *p* = 0.68 | *Est*. = 0.06  *SE =* 0.06  *t =* 0.97  *p* = 0.33 | *Est*. = -0.19  *SE =* 0.07  *t =* -2.76  *p* = 0.01 | *Est*. = -0.03  *SE =* 0.07  *t =* -0.43  *p* = 0.66 | *Est*. = 0.02  *SE =* 0.06  *t =* 0.29  *p* = 0.77 |
| Age^2^  *(standardized)* | *NA* | *Est*. = -0.10  *SE =* 0.01  *t =* -7.76  *p* < 0.001 | *NA* | *NA* | *NA* |
| Facility  *(reference = sanctuary)* | *Est*. = 0.29  *SE =* 0.09  *t =* 3.18  *p* < 0.01 | *Est*. = 0.29  *SE =* 0.09  *t =* 3.19  *p* < 0.01 | *Est*. = 0.17  *SE =* 0.10  *t =* 1.67  *p* = 0.10 | *Est*. = 0.17  *SE =* 0.10  *t =* 1.71  *p* = 0.09 | *Est*. = 0.30  *SE =* 0.09  *t =* 3.32  *p* < 0.01 |
| Age*Facility | *Est*. = 0.21  *SE =* 0.07  *t =* 3.16  *p* < 0.01 | *Est*. = 0.26  *SE =* 0.07  *t =* 3.82  *p* < 0.001 | *Est*. = 0.37  *SE* = 0.07  *t =* 5.21  *p* < 0.001 | *Est*. = 0.33  *SE =* 0.07  *t =* 4.70  *p* < 0.001 | *Est*. = 0.21  *SE =* 0.07  *t =* 3.12  *p* < 0.01 |
| Number of biomarkers | *Est*. = 0.03  *SE* < 0.01  *t =* 11.12  *p* < 0.001 | *Est*. = 0.02  *SE* < 0.01  *t =* 11.44  *p* < 0.001 | *Est*. = 0.01  *SE* < 0.01  *t =* 2.43  *p* = 0.02 | *Est*. = 0.03  *SE* < 0.01  *t =* 8.36  *p* < 0.001 | *Est*. = 0.03  *SE* < 0.01  *t =* 11.52  *p* < 0.001 |

***Sex***

We modeled the impact of sex on dysregulation separately in each population (sanctuary versus laboratory). The Ngamba dataset comprised 392 datapoints: 231 from females and 161 from males. The laboratory dataset comprised 3,351 datapoints: 2,015 from females and 1,336 from males. Raw *D_M_* scores in Figure S4 indicate that females at both sites show generally greater dysregulation compared to males, but while the sex difference decreases with age at the sanctuary, it increases with age at the laboratories. Model-predicted values are shown in Figure 2, and model effects are presented in Table S4.

*
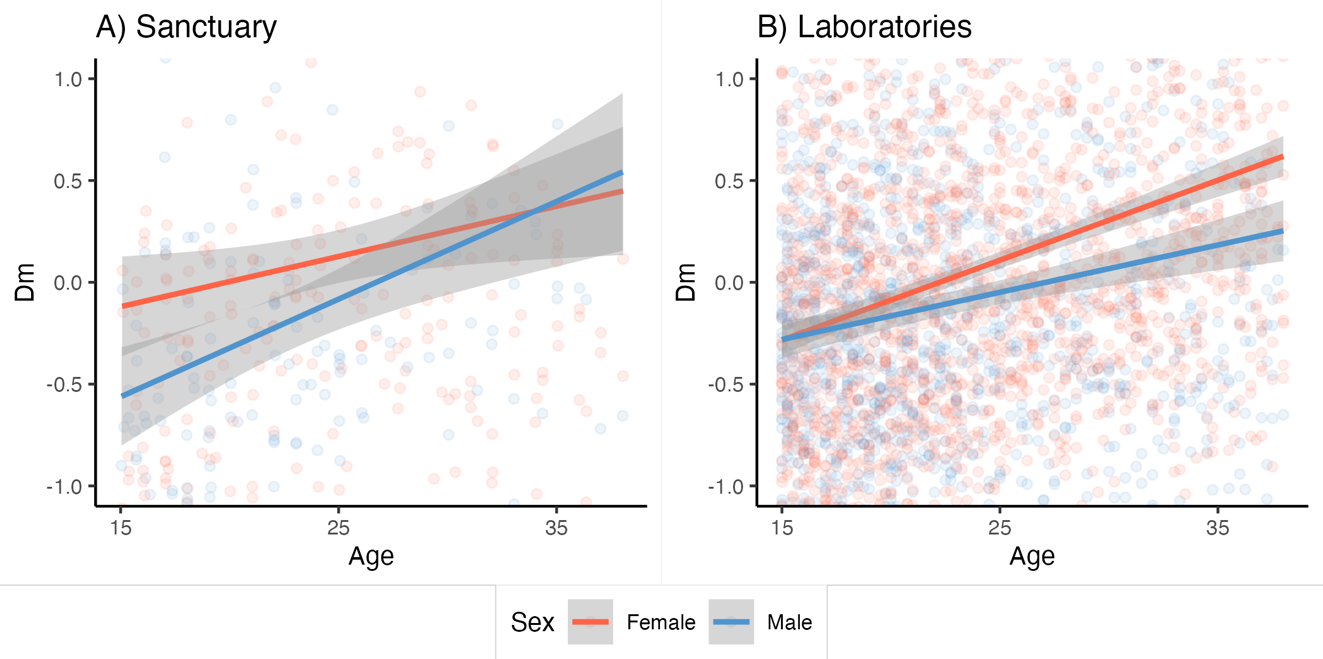
*

**Figure S4. Age-related change in *D_M_*, split by *sex* at the A) sanctuary and B) laboratories.** *D_M_* is log transformed and z-scored to one standard deviation. Lines indicate linear fit; ribbons indicate 95% confidence intervals.

**Table S4. Effects of sex models.** Parameters are from full models.

|  | **Sanctuary** | **Laboratories** |
| --- | --- | --- |
| Age  *(standardized)* | *Est*. = 0.14  *SE =* 0.06  *t =* 2.25  *p* = 0.03 | *Est*. = 0.23  *SE =* 0.02  *t =* 9.59  *p* < 0.001 |
| Sex  *(reference = female)* | *Est*. = -0.25  *SE =* 0.10  *t =* -2.64  *p* = 0.01 | *Est*. = -0.13  *SE =* 0.07  *t =* -1.94  *p* = 0.05 |
| Age*Sex | *Est*. = 0.15  *SE =* 0.10  *t =* 1.52  *p* = 0.13 | *Est*. = 0.04  *SE =* 0.04  *t =* 0.95  *p* = 0.34 |
| Number of biomarkers | *Est*. = 0.04  *SE* = 0.01  *t =* 6.12  *p* < 0.001 | *Est*. = 0.02  *SE* < 0.01  *t =* 9.45  *p* < 0.001 |
| Some outdoors / chow *(reference = outdoors / mixed diet)* | *NA* | *Est*. = -0.03  *SE =* 0.09  *t =* -0.35  *p* = 0.73 |
| Indoors / chow *(reference = outdoors / mixed diet)* | *NA* | *Est*. = -0.06  *SE =* 0.08  *t =* -0.72  *p* = 0.47 |

***Body system***

We also modeled body system separately by facility (sanctuary versus laboratory). To ensure that there were a sufficient number of biomarkers representing a given body system for a chimpanzee at a given time point, we required at least five biomarkers for that system. We therefore removed cardiometabolic biomarkers from this analysis due to low sample size (only 64 datapoints from Ngamba, and only 91 from the laboratories, all of which came from a single site). The final Ngamba dataset for this analysis comprised 1,143 datapoints: 375 from the circulatory system, and 384 each from the liver and renal and immune system. The laboratory dataset comprised 8,121 datapoints: 2,725 from the circulatory system, 3,104 from the liver and renal system, and 2,292 from the immune system. Raw *D_M_* scores in Figure S5 indicate that dysregulation is steepest for liver and renal biomarkers across sites, and similarly steep for immune biomarkers at Ngamba. Model-predicted values are shown in Figure 3, and model effects are presented in Table S5.

***
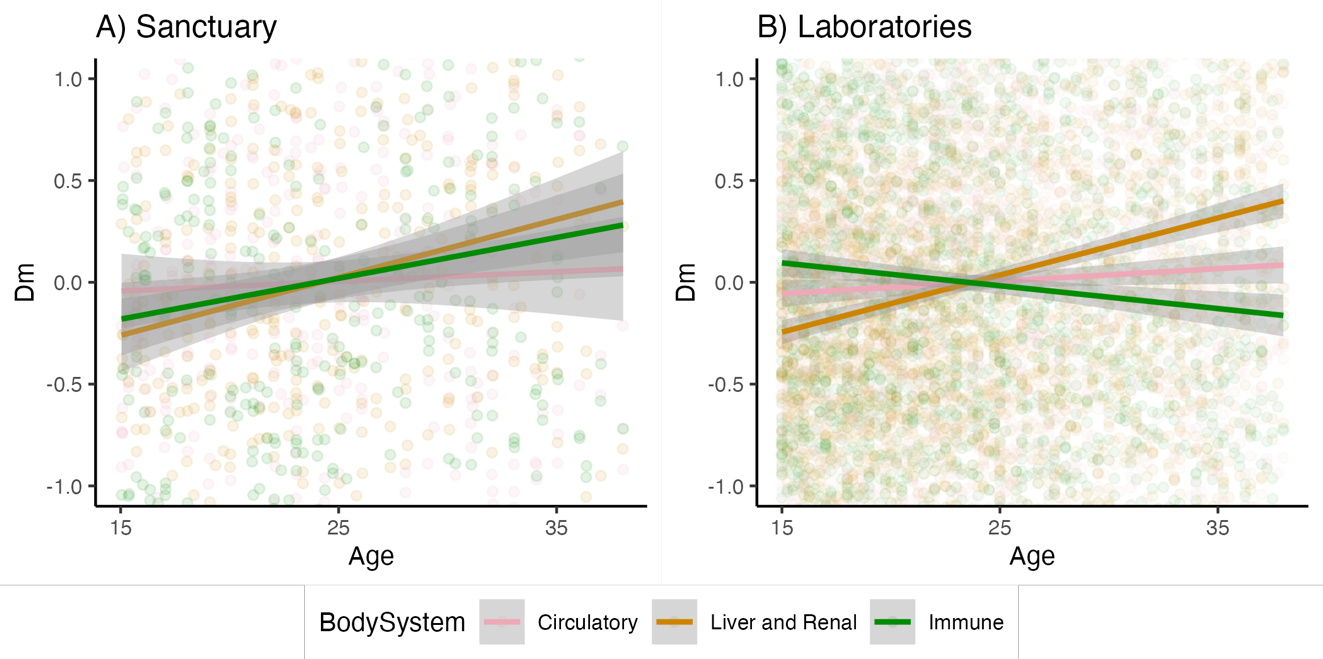
***

**Figure S5. Age-related change in *D_M_*, split by *body system* at the A) sanctuary and B) laboratories.** *D_M_* is log transformed and z-scored to one standard deviation. Lines indicate linear fit; ribbons indicate 95% confidence intervals. Cardiometabolic biomarkers were removed from body system analyses due to low sample size.

**Table S5. Effects of body system models.** Parameters are from full models.

|  | **Sanctuary** | **Laboratories** |
| --- | --- | --- |
| Age  *(standardized)* | *Est*. = -0.16  *SE =* 0.06  *t =* -2.65  *p* = 0.01 | *Est*. = 0.07  *SE =* 0.02  *t =* 3.64  *p* < 0.01 |
| Liver and renal system *(reference = circulatory)* | *Est*. = -0.19  *SE =* 0.08  *t =* -2.28  *p* = 0.02 | *Est*. = -0.30  *SE =* 0.05  *t =* -6.61  *p* < 0.001 |
| Immune system *(reference = circulatory)* | *Est*. = 0.30  *SE =* 0.10  *t =* 3.01  *p* =< 0.01 | *Est*. = 0.02  *SE =* 0.03  *t =* 0.77  *p* = 0.44 |
| Age*Liver and renal | *Est*. = 0.12  *SE =* 0.07  *t =* 1.76  *p* = 0.08 | *Est*. = 0.13  *SE =* 0.02  *t =* 5.36  *p* < 0.001 |
| Age*Immune | *Est*. = 0.11  *SE =* 0.07  *t =* 1.59  *p* = 0.11 | *Est*. = -0.11  *SE =* 0.03  *t =* -4.32  *p* < 0.001 |
| Number of biomarkers | *Est*. = 0.06  *SE* = 0.02  *t =* 4.19  *p* < 0.001 | *Est*. = 0.04  *SE* < 0.01  *t =* 7.75  *p* < 0.001 |
| Some outdoors / chow *(reference = outdoors / mixed diet)* | *NA* | *Est*. = -0.06  *SE =* 0.06  *t =* -0.95  *p* = 0.35 |
| Indoors / chow *(reference = outdoors / mixed diet)* | *NA* | *Est*. = -0.01  *SE =* 0.06  *t =* -0.12  *p* = 0.90 |

**3. Additional analyses**

In addition to the primary analyses reported in the main text, we also conducted several checks of the robustness of our results, since dysregulation scores can be sensitive to selection of reference samples and biomarker composition [2,4].

***Full laboratory age range***

In our main analyses, we only used data up to age 38 years for comparability across groups, given that the sanctuary data only spanned chimpanzees in this range. We additionally assessed the full laboratory dataset—i.e., with no age cutoff—to investigate whether patterns of age-related dysregulation in this population changed across adulthood. We also included an *age^2^* term in this analysis, to determine whether change in *D_M_* accelerates exponentially late in life as in some human groups. This extended dataset included 332 adult chimpanzees (194 females and 138 males) ranging in age from 15-58.25 years (mean=24.7) for a total of N=3,579 chimpanzee datapoints. In the full laboratory dataset, *D_M_* increased until approximately age 35, and plateaued until 45, before decreasing during later adulthood (Figure S6). However, it is important to note that the confidence band increases drastically during the period of senescence, reflecting the low sample size in this age range, and potentially a survivorship bias. Indeed, there were only N=45 datapoints above age 45, and most came from the “inside, chow only” laboratory population. Removing this site resulted in a plateau until end of life. Notably, when we allowed age to vary non-linearly, *D_M_* also decreased after young adulthood in the Ngamba sample (with a smaller age range).

Results of the full laboratory dataset analysis were similar to the main *facility analysis* (see Table S3 for model results and Table 3 for age slopes). Relative to the base model described in the main text, including *age^2^* improved model fit [χ2=55.91, df=1, p<0.001], but the *age^2^* coefficient was surprisingly *negative* [ß=-0.11; p<0.001], indicating that the pace of dysregulation decelerated during older age in this sample. As in the main analysis, including *facility* improved fit [χ2= 8.15, df=1, p<0.01]: *D_M_* was still higher overall at US laboratories. The addition of *age*facility* also improved model fit [χ2=13.95, df=1, p<0.001], and *D_M_* still increased with age faster at the laboratories (p<0.001). We assessed the effect of age on *D_M_* in the full laboratory dataset by extracting the standard effect coefficient for age from a linear mixed effects model controlling only for *subject identity*, *number of biomarkers*, and laboratory *site*. The slope for the full laboratory sample (0.24) was exactly the same as that of the truncated sample, showing that dysregulation does not accelerate during older adulthood here as it does in humans.


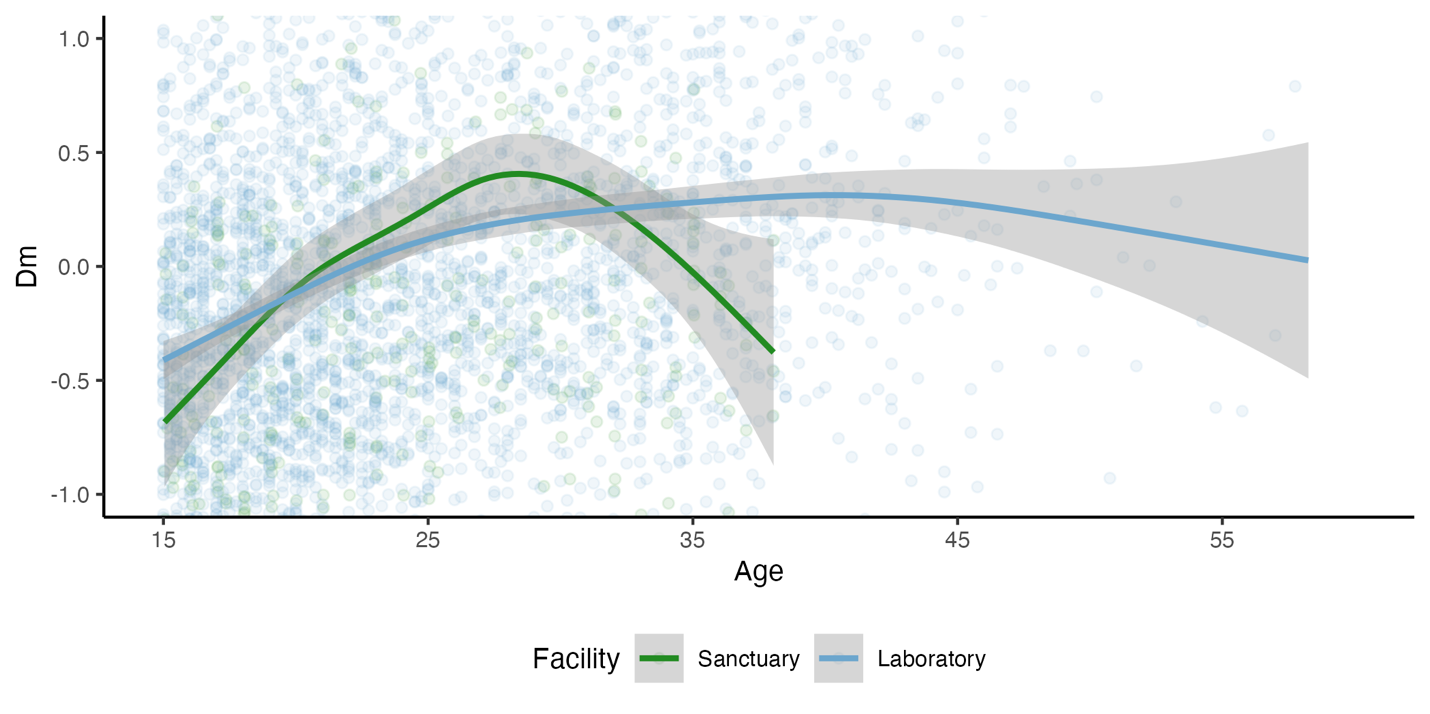


**Figure S6. Age-related change in *D_M_*, split by *facility* (full laboratory sample up to age 58.25).** *D_M_* is log transformed and z-scored to one standard deviation. Lines indicate gam fit; ribbons indicate 95% confidence intervals. Note small sample at laboratories after age 45.

***Pooled reference sample***

Our main analyses also used site-specific reference samples to allow for possible differences in physiology among young adults. Here we use the primary dataset but apply a pooled reference sample (combining healthy young adults across all four sites) to directly compare rates of aging across sites from a common starting point (see Table S3 for model results and Table 3 for age slopes). We removed TC/HDL at MD Anderson for being highly correlated to *D_M_,* but this did not alter our findings. Adding *facility* did not improve model fit relative to the base model [χ2=1.31, df=1, p=0.25]. But, adding *age*facility* did [χ2=26.26, df=1, p<0.001]: *D_M_* still increased with age faster at the laboratories (p<0.001). We assessed the effect of age on *D_M_* in the pooled reference dataset by extracting the standard effect coefficient for age in a separate model for each facility. Age slopes were lower when using a pooled sample, as compared to when using site-specific samples in the main analysis, for the laboratories (0.19) and especially for Ngamba (0.03, but no different from zero). This implies that age effects are sensitive to reference sample selection and additionally that young adult baselines are different across facilities.

***Common biomarkers***

Our main analyses used all available biomarkers at each site to maximize sample size. Because the number and type of biomarkers can influence results, we re-ran with a set of 25 matched biomarkers which were available at all four sites (Table 2). We excluded six observations at Ngamba for having fewer than five available biomarkers. Again, we found very similar model results (Table S3; Table 3). Adding *facility* did not improve model fit [χ2=1.54, df=1, p=0.22], but adding *age*facility* did [χ2=20.90, df=1, p<0.001], and *D_M_* still increased with age faster at the laboratories (p<0.001). We again assessed the effect of age on *D_M_* in the common biomarker dataset by extracting the standard effect coefficient for age in a separate model for each facility. Compared to the primary dataset which used all available biomarkers, this analysis produced a slightly shallower age slope at Ngamba (0.16) but a much steeper slope at the laboratories (0.30). Age effects therefore also appear mildly sensitive to biomarker selection.

***Alternative overweight cutoff***

As discussed above, we excluded overweight chimpanzees from healthy reference samples. In our primary analyses, we used overweight cutoffs originally defined by Videan et al. [3] using a population of laboratory-housed chimpanzees. These cutoffs removed a substantial proportion of both males and females from laboratory references in the current study (Figure S1). We did one last check to make sure this was not greatly impacting our results. We re-ran the main analysis using a less conservative approach which excluded only the top 20% of weight for each sex (>68.0 kg for females and > 70.0 kg for males). By these standards, 99% of observations (113/114) from young adults at Ngamba were included in the healthy reference sample, while 55% (709/1,286) were at the laboratories.

Results were nearly identical to the main analysis. Relative to the base model, model fit was improved with inclusion of *facility* [χ2=9.40, df=1, p<0.01], and dysregulation scores were higher at laboratories. Addition of an *age*facility* interaction further improved fit [χ2=9.06, df=1, p<0.01], and dysregulation progressed faster at laboratories (p<0.01). As above, we assessed the effect of age on *D_M_* in the alternative overweight cutoff dataset by extracting the standard effect coefficient for age in a separate model for each facility. Age slopes were again very close to the primary analysis which used a more conservative cutoff: 0.17 at the sanctuary and 0.23 at the laboratories.

**4. Supplemental references**

1. Cohen AA, Milot E, Yong J *et al.* A novel statistical approach shows evidence for multi-system physiological dysregulation during aging. *Mech Ageing Dev* 2013;**134**:110–7.

2. Cohen AA, Li Q, Milot E *et al.* Statistical Distance as a Measure of Physiological Dysregulation Is Largely Robust to Variation in Its Biomarker Composition. Koomen JM (ed.). *PLOS ONE* 2015;**10**:e0122541.

3. Videan EN, Fritz J, Murphy J. Development of guidelines for assessing obesity in captive chimpanzees (*Pan troglodytes*). *Zoo Biol* 2007;**26**:93–104.

4. Dansereau G, Wey TW, Legault V *et al.* Conservation of physiological dysregulation signatures of aging across primates. *Aging Cell* 2019;**18**:e12925.
